# Neoadjuvant atezolizumab plus chemotherapy in gastric and gastroesophageal junction adenocarcinoma: the phase 2 PANDA trial

**DOI:** 10.1038/s41591-023-02758-x

**Published:** 2024-01-08

**Authors:** Yara L. Verschoor, Joris van de Haar, José G. van den Berg, Johanna W. van Sandick, Liudmila L. Kodach, Jolanda M. van Dieren, Sara Balduzzi, Cecile Grootscholten, Marieke E. IJsselsteijn, Alexander A. F. A. Veenhof, Koen J. Hartemink, Marieke A. Vollebergh, Adham Jurdi, Shruti Sharma, Erik Spickard, Emilia C. Owers, Annemarieke Bartels-Rutten, Peggy den Hartog, Noel F. C. C. de Miranda, Monique E. van Leerdam, John B. A. G. Haanen, Ton N. Schumacher, Emile E. Voest, Myriam Chalabi

**Affiliations:** 1 Department of Gastrointestinal Oncology, Netherlands Cancer Institute – Antoni van Leeuwenhoek Hospital, Amsterdam, the Netherlands; 2 Department of Molecular Oncology and Immunology, Netherlands Cancer Institute – Antoni van Leeuwenhoek Hospital, Amsterdam, the Netherlands; 3 Oncode Institute, Amsterdam, the Netherlands; 4 Department of Pathology, Netherlands Cancer Institute – Antoni van Leeuwenhoek Hospital, Amsterdam, the Netherlands; 5 Department of Surgery, Netherlands Cancer Institute – Antoni van Leeuwenhoek Hospital, Amsterdam, the Netherlands; 6 Biometrics department, Netherlands Cancer Institute – Antoni van Leeuwenhoek Hospital, Amsterdam, the Netherlands; 7 Department of Pathology, Leiden University Medical Center, Leiden, the Netherlands; 8 Department of Medical Oncology, Netherlands Cancer Institute – Antoni van Leeuwenhoek Hospital, Amsterdam, the Netherlands; 9 Natera, Inc, Austin, TX USA; 10 Department of Nuclear Medicine, Netherlands Cancer Institute – Antoni van Leeuwenhoek Hospital, Amsterdam, the Netherlands; 11 Department of Radiology, Netherlands Cancer Institute – Antoni van Leeuwenhoek Hospital, Amsterdam, the Netherlands; 12 Department of Gastroenterology and Hepatology, Leiden University Medical Center, Leiden, the Netherlands; 13 Department of Medical Oncology, Leiden University Medical Center, Leiden, the Netherlands; 14 Oncology Service, Centre Hospitalier Universitaire Vaudois, Lausanne, Switzerland; 15 Department of Hematology, Leiden University Medical Center, Leiden, the Netherlands

**Keywords:** Phase II trials, Tumour immunology, Biomarkers, Gastric cancer

## Abstract

Gastric and gastroesophageal junction (G/GEJ) cancers carry a poor prognosis, and despite recent advancements, most patients die of their disease. Although immune checkpoint blockade became part of the standard-of-care for patients with metastatic G/GEJ cancers, its efficacy and impact on the tumor microenvironment (TME) in early disease remain largely unknown. We hypothesized higher efficacy of neoadjuvant immunotherapy plus chemotherapy in patients with nonmetastatic G/GEJ cancer. In the phase 2 PANDA trial, patients with previously untreated resectable G/GEJ tumors (*n* = 21) received neoadjuvant treatment with one cycle of atezolizumab monotherapy followed by four cycles of atezolizumab plus docetaxel, oxaliplatin and capecitabine. Treatment was well tolerated. There were grade 3 immune-related adverse events in two of 20 patients (10%) but no grade 4 or 5 immune-related adverse events, and all patients underwent resection without treatment-related delays, meeting the primary endpoint of safety and feasibility. Tissue was obtained at multiple time points, allowing analysis of the effects of single-agent anti-programmed cell death ligand 1 (PD-L1) and the subsequent combination with chemotherapy on the TME. Twenty of 21 patients underwent surgery and were evaluable for secondary pathologic response and survival endpoints, and 19 were evaluable for exploratory translational analyses. A major pathologic response (≤10% residual viable tumor) was observed in 14 of 20 (70%, 95% confidence interval 46–88%) patients, including 9 (45%, 95% confidence interval 23–68%) pathologic complete responses. At a median follow-up of 47 months, 13 of 14 responders were alive and disease-free, and five of six nonresponders had died as a result of recurrence. Notably, baseline anti-programmed cell death protein 1 (PD-1)^+^CD8^+^ T cell infiltration was significantly higher in responders versus nonresponders, and comparison of TME alterations following anti-PD-L1 monotherapy versus the subsequent combination with chemotherapy showed an increased immune activation on single-agent PD-1/L1 axis blockade. On the basis of these data, monotherapy anti-PD-L1 before its combination with chemotherapy warrants further exploration and validation in a larger cohort of patients with nonmetastatic G/GEJ cancer. ClinicalTrials.gov registration: NCT03448835.

## Abstract (web summary)

A neoadjuvant treatment regimen of anti-PD-L1 monotherapy followed by anti-PD-L1 plus chemotherapy was well tolerated and led to a major pathologic response rate of 70% in patients with resectable gastric or gastroesophageal junction adenocarcinoma.

## Main

G/GEJ cancers represent a principal cause of mortality, ranking as the fourth most common cause of cancer-related death worldwide^1^. Despite improvement of survival through implementation of perioperative chemotherapy and chemoradiotherapy, overall prognosis remains poor, with a 5-year survival of patients with locally advanced G/GEJ adenocarcinoma of less than 50% (refs. ^2,3^). In the pivotal FLOT4 study, comparing FLOT chemotherapy (docetaxel, oxaliplatin, leucovorin and fluorouracil) with the then standard-of-care—epirubicin, cisplatin, fluorouracil/capecitabine— FLOT led to significant improvement in overall survival (OS, 35 versus 50 months) and disease-free survival (DFS, 30 versus 18 months)^3,4^. Moreover, higher rates of pathologic complete responses (pCR, 16%) and near-complete responses (21%) were observed in FLOT-treated patients compared to epirubicin, cisplatin, fluorouracil/capecitabine (6% and 17%, respectively) in the FLOT4-AIO study^4^. On the basis of these data, FLOT has become standard-of-care.

Over the past decade, immune checkpoint blockade (ICB) has emerged as an effective and promising therapeutic approach in a variety of malignancies. PD-L1, an inhibitory checkpoint molecule, has been shown to be upregulated in gastric cancer^5,6^, and its expression negatively correlates with prognosis and survival^7,8^. In patients with advanced or metastatic G/GEJ cancer, randomized trials have shown improved clinical outcomes after anti-PD-L1 with chemotherapy compared to chemotherapy alone, leading to approval of this regimen as first-line treatment^9–11^. Although some level of activity was observed in patients with low or absent PD-L1 expression, the largest effect size was in tumors expressing PD-L1 with combined positive scores (CPS) varying from ≥1 to ≥5, leading to approval by the European Medicines Agency contingent on PD-L1 CPS^9,10^. Importantly, anti-programmed cell death protein 1 (PD-1) has also been shown to improve survival in patients with stage II and III disease in the Checkmate-577 study, in which DFS was significantly higher in patients treated with adjuvant nivolumab compared to placebo in patients undergoing surgery after chemoradiotherapy for esophageal and GEJ adenocarcinoma, regardless of PD-L1 expression^11^. Overall, these studies demonstrated efficacy of anti-PD-1 in G/GEJ cancer, yet the inconsistent association between PD-L1 expression and benefit from anti-PD-1 indicates that efficacy is probably determined by further factors and that predictive biomarkers are urgently needed.

Neoadjuvant ICB has been shown to result in remarkable pathologic responses in several tumor types, including melanoma, lung cancer, bladder cancer and colorectal cancer. In addition, pathologic responses to ICB are highly correlated with improved survival^12–18^. The high response rate to neoadjuvant ICB may be attributed to the presence of a larger amount of antigen with which checkpoint inhibition can synergize^19^. Also, it may be speculated that, in case of tumor-draining lymph node dissection, ICB before surgery may benefit from the presence of a larger pool of tumor-reactive T cells. Furthermore, recent studies have shown the predictive value of interferon (IFN)-γ signatures in the pathologic response to neoadjuvant immunotherapy in melanoma, which has led to interventional studies allocating patients to different neoadjuvant regimens according to IFNγ signatures^20^. These data highlight the importance of predictive markers in personalization of neoadjuvant therapies.

Although there is increasing evidence for the immune-potentiating effects of combined ICB plus chemotherapy^21,22^, the order in which to administer these therapies to achieve optimal antitumor efficacy remains to be elucidated. Preclinical studies have demonstrated a higher increase in CD8^+^ T cell infiltration (TCI) and improved antitumor responses when anti-PD-L1 is given before the start of chemotherapy, as compared to either concomitant or subsequent PD-L1 blockade^23^. In early triple-negative breast cancer, anti-PD-1 monotherapy given before chemotherapy was associated with a higher pCR rate than concomitant anti-PD-1 plus chemotherapy^24,25^. These data indicate that ICB before chemotherapy may prime the TME and thereby elicit enhanced antitumor efficacy. In the current study, we aimed to evaluate the immune-modulating effects of PD-L1 blockade alone by administering monotherapy atezolizumab before its combination with chemotherapy.

Here we present results from the PANDA study, investigating the safety, efficacy and immunologic correlates of atezolizumab plus chemotherapy in patients with resectable, nonmetastatic G/GEJ adenocarcinoma.

**Extended Data Fig. 1:**
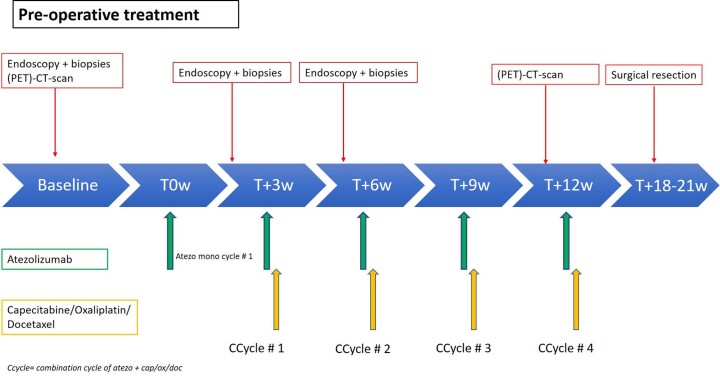
Study design. At baseline, all patients underwent FDG-PET and CT-scan as well as esophagoduodenoscopy with representative biopsies from tumor and normal tissue. Patients were treated with one cycle of atezolizumab 1200 mg monotherapy, followed by four combination cycles of atezolizumab 1200 mg, docetaxel 50 mg/m2, oxaliplatin 100 mg/m2 and capecitabine 850 mg. Representative biopsies from tumor and normal tissue were obtained after monotherapy atezolizumab and after the first combination cycle. Surgery was performed 6–9 weeks after the last treatment cycle and tissue was obtained from surgical resection specimens.

In the PANDA study (NCT03448835), patients received one cycle of monotherapy atezolizumab (1,200 mg) followed by four cycles of atezolizumab (1,200 mg) combined with docetaxel (50 mg m^−^^2^), oxaliplatin (100 mg m^−^^2^) and capecitabine (850 mg m^−^^2^) (DOC). Tumor biopsies were obtained through endoscopy at baseline, after monotherapy atezolizumab and after the first combination treatment. Surgery was performed 6–9 weeks after the last treatment cycle, and tissue was obtained from surgical resection specimens (Extended Data Fig. 1). The primary objective was safety and feasibility. Secondary objectives included efficacy assessed by histopathologic regression, changes in the TME and clinical outcomes. Pathologic response was defined as ≤10% residual viable tumor (RVT), consisting of major pathologic response (MPR, ≤10% RVT) and pathologic complete response (pCR, 0% RVT)^17,26,27^. An important purpose of this study design was to allow separate dissection of the effects of PD-L1 blockade alone and the subsequent combination with chemotherapy on the TME, as well as their association with clinical response.

## Results

### Patient characteristics

**Extended Data Fig. 2:**
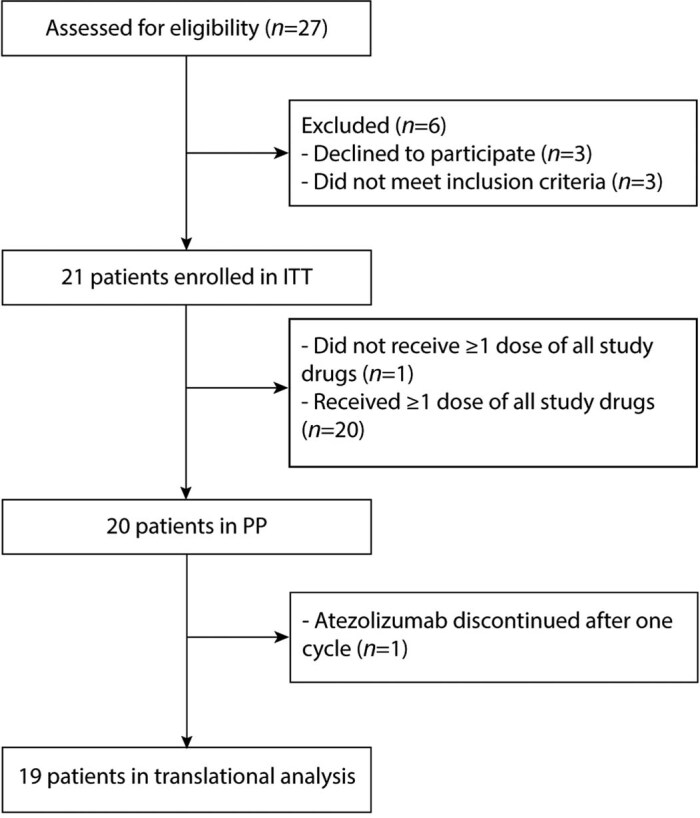
Consort diagram. Flowchart of patients included in the PANDA study. The intention-to-treat (ITT) population was defined as all patients enrolled in the study. The per-protocol (PP) population was defined as all patients who received at least one dose of the study drugs (atezolizumab, docetaxel, oxaliplatin and capecitabine).

From 12 April 2018 to 14 May 2021, a total of 21 patients were enrolled. Baseline patient and tumor characteristics are summarized in Table 1. The median age was 62 years (range 46–76), and 19 of 21 (90%) patients were male. On the basis of pretreatment staging by computed tomography (CT) and/or fluorodeoxyglucose-positron emission tomography (FDG-PET) scan combined with endoscopic ultrasound, 17 of 21 (81%) patients had a cT3 or cT4a tumor and 16 of 21 (76%) had cN+ disease (Extended Data Table 1). Three patients had a mismatch repair deficient (dMMR) tumor, and one patient an Epstein–Barr virus (EBV)^+^ tumor. One patient with a dMMR tumor died shortly after atezolizumab monotherapy because of external factors unrelated to the study treatment. Twenty patients underwent surgery and were evaluable in the per-protocol (PP) population for safety and secondary efficacy endpoints according to the study protocol (Extended Data Fig. 2).

**Table 1:** Baseline patient and tumor characteristics

	Overall (*n* = 21)
**Age, median (range)**	62 (46–76)
**Sex**	
Male	19 (90%)
Female	2 (10%)
**ECOG performance status**	
0	15 (71%)
1	6 (29%)
**Primary tumor location**	
Gastric	5 (24%)
GEJ Siewert type 1	1 (5%)
GEJ Siewert type 2	9 (43%)
GEJ Siewert type 3	6 (29%)
**Clinical T stage**	
T2	4 (19%)
T3	15 (71%)
T4a	2 (10%)
**Clinical N stage**	
N0	5 (24%)
N1	11 (52%)
N2	4 (19%)
N3	1 (5%)
**AJCC clinical stage**^68^	
IB	2 (10%)
IIA	4 (19%)
IIB	9 (43%)
IIIA	5 (24%)
IIIB	1 (5%)
**Lauren classification**	
Intestinal	16 (76%)
Diffuse/mixed	4 (19%)
Indeterminate	1 (5%)
**MMR status**	
MMR proficient	18 (86%)
MMR deficient^a^	3 (14%)
**PD-L1 CPS**^b^	
CPS ≥ 1	18 (90%)
CPS ≥ 5	14 (70%)
CPS ≥ 10	4 (20%)

AJCC, American Joint Committee on Cancer.

^a^One patient with a dMMR tumor did not undergo surgery and was excluded from efficacy and translational endpoints.

^b^PD-L1 CPS was available for 20 patients.

### Safety

Overall, treatment was well tolerated, and the study met its primary endpoint of safety and feasibility. Immune-related adverse events (irAEs) of any grade occurred in 11 of 20 (55%) patients, and three grade 3 irAEs were observed in two (10%) patients, consisting of hepatitis, headache and diarrhea (Table 2). There were no grade 4 or 5 irAEs.

**Table 2:** Immune-related adverse events (*n* = 20)

	Number of patients with event (%)		
	Grade 1	Grade 2	Grade 3
**Any immune-related adverse event**	**9 (45%)**	**7 (35%)**	**2 (10%)**
Infusion-related reaction	2 (10%)	3 (15%)	–
Fatigue	4 (20%)	–	–
Fever	–	4 (20%)	–
Arthralgia	1 (5%)	1 (5%)	–
Hypothyroidism	1 (5%)	1 (5%)	–
Diarrhea	–	–	1 (5%)
Headache	–	–	1 (5%)
Hepatitis	–	–	1 (5%)
Photosensitivity	1 (5%)	–	–
Rash maculopapular	1 (5%)	–	–

All AEs that were deemed at least possibly related to atezolizumab are shown in this table. This is regardless of the possible relationship with chemotherapy.

In one patient, grade 3 immune-related hepatitis and meningitis were suspected on the basis of elevated liver enzymes and headache following monotherapy atezolizumab. Although liver biopsy and cerebrospinal fluid analysis failed to confirm these diagnoses, high-dose steroids and mycophenolate mofetil were started, with complete resolution of both AEs. Atezolizumab was discontinued, and the patient received all cycles of neoadjuvant chemotherapy. One patient with grade 3 diarrhea had complete resolution of symptoms within 1 week with supportive treatment. Finally, one patient who was excluded from the PP population experienced grade 3 fatigue after monotherapy atezolizumab, and steroid treatment was initiated. This AE could not be followed up because of study unrelated death.

Chemotherapy was administered to all 20 patients. Chemotherapy dose delays (>7 days) occurred in one of 80 (1%) cycles in one (5%) patient, dose reductions were required in eight of 80 (10%) cycles in five (25%) patients, and omission of chemotherapeutic drugs occurred in three of 80 cycles (4%) in two (10%) patients (Extended Data Table 2). Grade 3 chemotherapy-related AEs were observed in four patients (20%, Extended Data Table 3) and consisted of febrile neutropenia (15%) and diarrhea (5%).

Twenty patients underwent surgery, all without treatment-related delays. One patient chose to postpone surgery for personal reasons. The median interval between the last study treatment and surgery was 6 weeks (range 5–13 weeks). Thirteen patients underwent transhiatal esophagectomy with gastric tube reconstruction and cervical anastomosis, six patients a total gastrectomy with Roux-and-Y reconstruction and one patient subtotal gastrectomy with Billroth II reconstruction. Surgical resection margins were tumor-free (R0) in 19 of 20 (95%) patients. One patient undergoing total gastrectomy for linitis plastica with a tumor-positive distal resection margin underwent adjuvant chemoradiotherapy.

No unexpected surgical complications were observed, and there were no intraoperative complications or surgery-related deaths. Surgery-related AEs of any grade were observed in 11 of 20 patients (55%), and grade 3 or 4 AEs were observed in 10 patients (50%) (Extended Data Table 4). Anastomotic leakage occurred in three of 20 (15%) patients; all three patients underwent esophagectomy with cervical anastomosis, and leakage was treated with stents (*n* = 2) or conservatively (*n* = 1).

### Neoadjuvant ICB plus chemo leads to high pathologic response rates

**Fig. 1:**
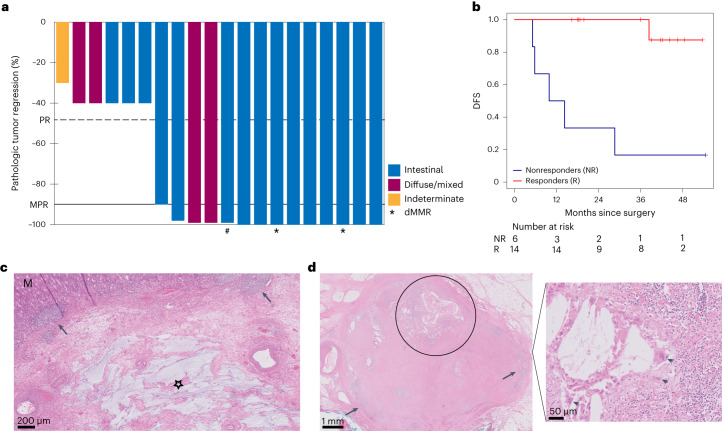
Pathologic responses and outcomes after neoadjuvant atezolizumab plus DOC chemotherapy. **a**, Percentage of pathologic regression shown per tumor. The black horizontal line depicts the demarcation for MPRs corresponding to 90% tumor regression. The dashed line demarcates PR (50% tumor regression). Colors of the bars represent different Lauren subtypes, the asterisks below bars indicate dMMR tumors, and the hash (#) indicates the patient with a pCR in the primary tumor but <1% RVT in lymph nodes. **b**, Kaplan–Meier plot of DFS for pathologic responders (red) versus nonresponders (blue) in patients evaluable for response in the PANDA trial. **c**, Posttreatment resection specimen from a patient with a pMMR GEJ tumor (cT3N1) who had a pCR. H&E stain showing normal mucosa (M) and complete regression of the adenocarcinoma, which was characterized by mucin lakes (star) and tertiary lymphoid structures (arrowheads). **d**, Posttreatment H&E stains of a resected lymph node from a patient with pretreatment clinical N3 stage. Left, some preexistent lymphoid tissue (arrowheads) and complete tumor regression characterized by cholesterol clefts (circle). Right, multinucleated giant cells (arrowheads).

Fourteen of 20 (70%, 95% confidence interval (CI) 46–88%) patients had a pathologic response, all consisting of an MPR with ≤10% RVT (Mandard tumor regression grading (TRG)1 or 2), including nine of 20 (45%, 95% CI 23–68%) pathologic complete responses (Mandard TRG1). Among 18 patients with a mismatch repair proficient (pMMR) tumor, an MPR was observed in 12 (67%, 95% CI 41–87%) patients, including seven (39%, 95% CI 17–64%) pCRs (Extended Data Table 5). Both patients with a dMMR tumor who underwent surgery had a pCR. The two patients with HER2+ tumors had a pCR and an MPR with 1% RVT. One patient with a pCR of the primary tumor but <1% RVT in lymph nodes was classified as having an MPR (Fig. 1a). Remarkably, pCR was not restricted to patients with pretreatment American Joint Committee on Cancer stage I and IIA tumors but was also observed in patients with stage IIB, IIIA and even IIIB tumors (Extended Data Table 1).

Tumor regression was characterized histologically by fibrosis, neuronal hyperplasia, influx of immune cells, acellular mucin pools and regional necrosis (Fig. 1c). Notably, resection specimens from multiple patients contained lymph nodes with evidence of histologic regression, including a patient with pretreatment clinical N3 stage whose resection specimen contained eight tumor-regressed lymph nodes without viable tumor cells (Fig. 1d). Pathologically assessed downstaging was evident in 13 of 20 patients. Furthermore, all six nonresponders (Mandard TRG3–5) displayed some pathologic regression, with 60–70% RVT. This included the patient with an EBV^+^ tumor, who had received only one cycle of atezolizumab.

When considering intestinal and diffuse/mixed subtypes separately, an MPR was observed in 12 of 15 (80%, 95% CI 52–96%) intestinal-type tumors, including nine of 15 (60%, 95% CI 32–84%) pCR. In the four tumors with a diffuse/mixed type histology, an MPR was observed in two (50%, 95% CI 7–93%) patients.

### Response assessment by CT and FDG-PET imaging

For the secondary endpoint of radiographic response, CT and/or FDG-PET imaging was performed before the last treatment cycle in 19 and 17 patients, respectively. Notably, among 19 patients with posttreatment CT scans, only one patient had a measurable target lesion according to Response Evaluation Criteria in Solid Tumors (RECIST) v.1.1, because primary tumors in hollow organs are not considered target lesions according to RECIST v.1.1, regardless of size. Therefore, response assessment was mostly descriptive. Of the 13 pathologic responders who underwent preoperative CT scans, 11 patients were described as having a decrease in tumor size, yet none of the nine patients with a pCR were radiographically assessed as complete responders.

In the 17 patients who underwent preoperative FDG-PET scans, the posttreatment maximum standardized uptake value (SUV_max_) was significantly different (*P* = 0.05) between responders (median 4.2, range 2.3–6.5) and nonresponders (median 5.7, range 4.5–5.8). Although ten of 12 pathologic responders were evaluated as (near-)complete responders, the remaining two responders (both pCR in the primary tumor) were assessed as partial responder and stable disease. In the five pathologic nonresponders who underwent FDG-PET, four patients were evaluated as responders on FDG-PET.

Metabolic response was also assessed by calculating the difference in total lesion glycolysis (ΔTLG) between baseline and posttreatment FDG-PET, which was shown to accurately predict pathologic response to neoadjuvant ICB in head and neck cancers^28^. However, among 17 FDG-PET evaluable patients in our cohort, 13 had a ΔTLG of −100%, including pathologic nonresponders, indicating the inaccuracy of this method in our cohort. In the remaining four patients, ΔTLG could not be calculated because the inability to accurately delineate the tumor impeded metabolic tumor volume (MTV) calculation (Supplementary Table 1).

These data are in line with the previously described underestimation of response to (neoadjuvant) immunotherapy by radiographic assessment across tumor types, highlighting the need for more accurate methods of response evaluation^17,29–31^.

### Association of pathologic response with outcome

**Extended Data Fig. 3:**
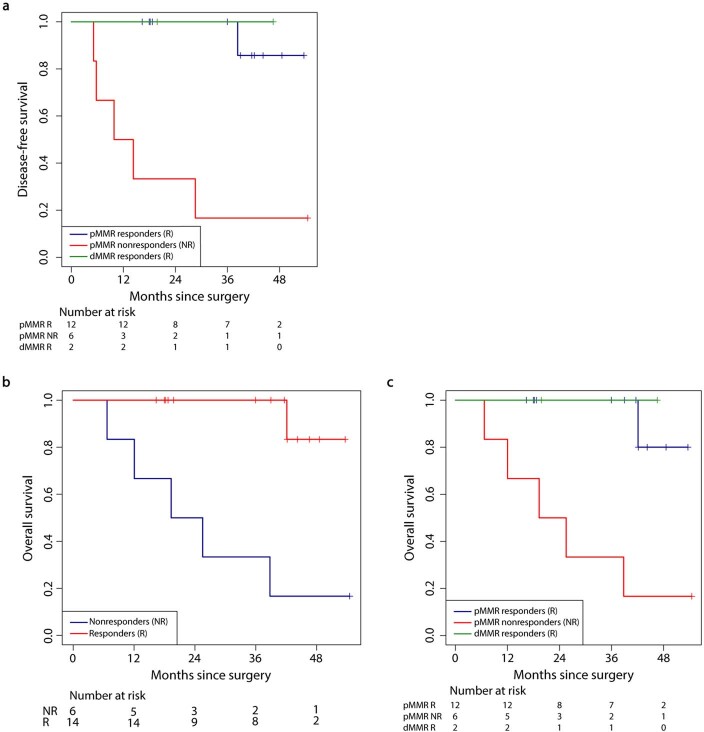
Kaplan-Meier plots of disease-free and overall survival for responders versus nonresponders. **a**, DFS for pMMR pathologic responders (blue) versus dMMR pathologic responders (green) versus pMMR pathologic nonresponders (red). **b**, Overall survival (OS) for pathologic responders (red) versus nonresponders (blue). **c**, OS for pMMR pathologic responders (blue) versus dMMR pathologic responders (green) versus pMMR pathologic nonresponders (red).

Clinical outcome showed a strong relationship with pathologic response, both secondary endpoints, after neoadjuvant therapy, as demonstrated by a significantly higher DFS (*P* = 0.0001, Fig. 1b) and OS (*P* = 0.0006, Extended Data Fig. 3) in responders compared to nonresponders. At the time of data cutoff on 24 April 2023, the median follow-up was 47 months (range 11–59), and 13 of 14 (93%) patients with a pCR or MPR were alive and disease-free. In contrast, only one of six nonresponders (16%) remained disease-free, and five of six (83%) patients developed disease recurrence (Fig. 1b).

Median DFS was not reached (95% CI 38—not reached), and DFS at 3 years was 73% (95% CI 55–97%). Disease recurrences in nonresponders occurred after a median follow-up of 10 months (range 5–29) after surgery, and nonresponding patients with disease recurrence died with a median survival after recurrence of 10 months (range 2–14).

The nonresponder who remained disease-free had an EBV^+^ tumor. One responder with a cT3N3 tumor at baseline who had a pCR developed recurrent disease in the brain at 38 months after surgery. A solitary brain lesion was resected and confirmed clonal relatedness to the primary tumor. The patient died 4 months after diagnosis of metastatic disease as a result of rapidly progressive brain metastases.

### Circulating tumor DNA is associated with response and DFS

**Extended Data Fig. 4:**
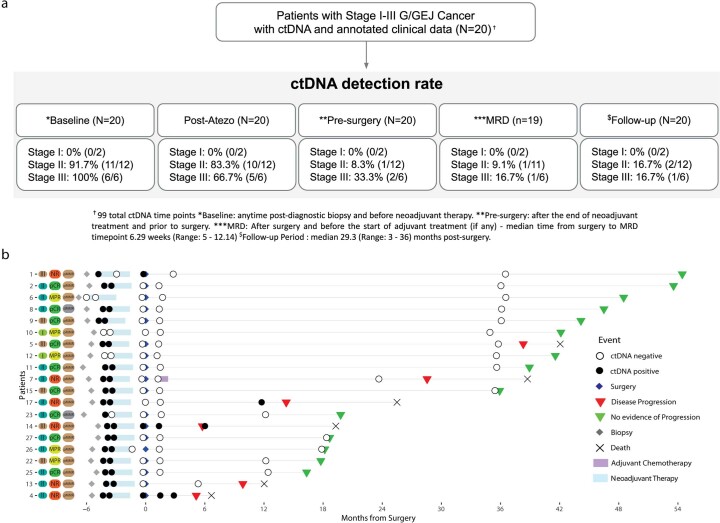
Cohort characteristics and ctDNA testing details. **a**, Flow diagram depicting an overview of the number of patients and plasma samples sorted by stage. **b**, Longitudinal representation of ctDNA results for analyzed samples (*n* = 20). Patients are ordered by decreasing clinical follow-up.

At baseline, circulating tumor DNA (ctDNA) could be detected in 85% (17 of 20) of patients across all stages, with 94.4% (17 of 18) of patients with tumors of stage II or higher (Extended Data Fig. 4). CtDNA status was also analyzed after monotherapy atezolizumab, before surgery, postsurgery (molecular residual disease) and during follow-up, with ctDNA positivity rates of 75% (15 of 20), 15% (3 of 20), 10.5% (2 of 19) and 15% (3 of 20), respectively.

**Fig. 2:**
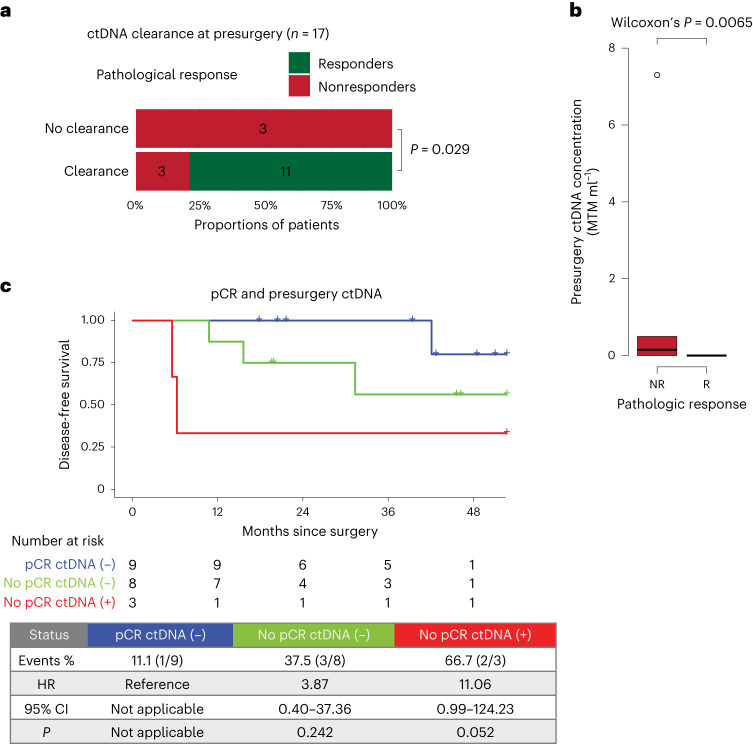
Association of ctDNA with pathologic response. **a**, Association between pathologic response and ctDNA clearance after all neoadjuvant treatment cycles and before surgery (*n* = 17). Significance was tested using a one-sided Fisher’s exact test. **b**, Average ctDNA concentration, in mean tumor molecules per milliliter of plasma (MTM per ml), across nonresponders (red, *n* = 6) and responders (green, *n* = 14). Box plots represent the median, 25th and 75th percentiles; the whiskers extend from the hinge to the largest value no farther than 1.5 × interquartile range (IQR) from the hinge. For comparison between nonresponders (red) and responders (green), significance was tested using a two-sided Wilcoxon rank-sum test. **c**, Kaplan–Meier plot of DFS stratified by combination of pathologic response (responders/nonresponders) and presurgery ctDNA status. Hazard ratios (HRs) and 95% CIs were calculated using the Cox proportional hazard model. *P* values were calculated using the two-sided log-rank test.

**Extended Data Fig. 5:**
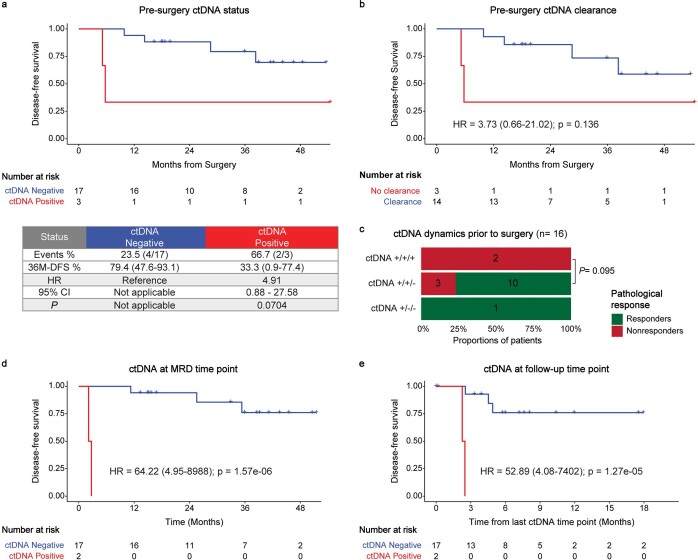
Association of ctDNA testing with clinical outcomes and pathologic response. **a**, Kaplan-Meier plots of DFS stratified by ctDNA-negative and ctDNA-positive status or **b**, ctDNA clearance and no clearance at the pre-surgery time point. HRs and 95% CIs were calculated using the Cox proportional hazard model. Significance was calculated using the two-sided log-rank test. **c**, Association between pathologic response and ctDNA dynamics from baseline, after monotherapy atezolizumab and before surgery (n = 16). Group allocation: ctDNA +/+/+: no clearance prior to surgery; ctDNA +/+/−: clearance prior to surgery; ctDNA +/−/−: clearance after monotherapy atezolizumab. Significance was tested using one-sided Fisher’s exact test. **d**,**e**, Kaplan-Meier plots of DFS stratified by ctDNA-negative and ctDNA-positive status at the MRD (**d**) or follow-up (**e**) time point post-surgery. To account for immortal time bias, a landmark analysis was performed 12 weeks after surgery (**d**) and from the last ctDNA follow-up time point after surgery (**e**). HRs and 95% CIs were calculated using the Cox Regression with Firth’s Penalized Likelihood hazard model. Significance was calculated using a two-sided log-rank test. MRD: Molecular Residual Disease.

After all neoadjuvant treatment cycles and before surgery, ctDNA was cleared in 11 of 11 responders, whereas three of six nonresponders remained positive (*P* = 0.029, Fig. 2a and Extended Data Fig. 5c). In addition, ctDNA levels were significantly higher in nonresponders than in responders (*P* = 0.0065, Fig. 2b). These data indicate an association between ctDNA and pathologic response to neoadjuvant treatment. Although ctDNA positivity and nonclearance at the presurgery time point were associated with an inferior DFS, this was not significant, probably because of the small sample size (Extended Data Fig. 5a,b). When considering pathologic response and presurgery ctDNA status simultaneously, ctDNA-positive nonresponders had a higher risk of recurrence than ctDNA-negative patients with a pCR (Fig. 2c). Furthermore, ctDNA positivity at the molecular residual disease and follow-up time points was associated with a 100% recurrence rate (Extended Data Fig. 5d,e).

### Biomarkers predictive of response to neoadjuvant ICB

With the exploratory aim of identifying potential biomarkers predictive of response, immunohistochemistry (IHC), RNA sequencing and whole-exome sequencing (WES) were performed on tumor biopsies to explore differences between responders (≤10% RVT, *n* = 14) and nonresponders (>10% RVT, *n* = 5). One patient who discontinued atezolizumab after the first cycle was excluded from exploratory translational analyses because this TME was not considered representative for a response to the study treatment. An overview of samples used for translational analyses is provided in Supplementary Fig. 1.

**Fig. 3:**
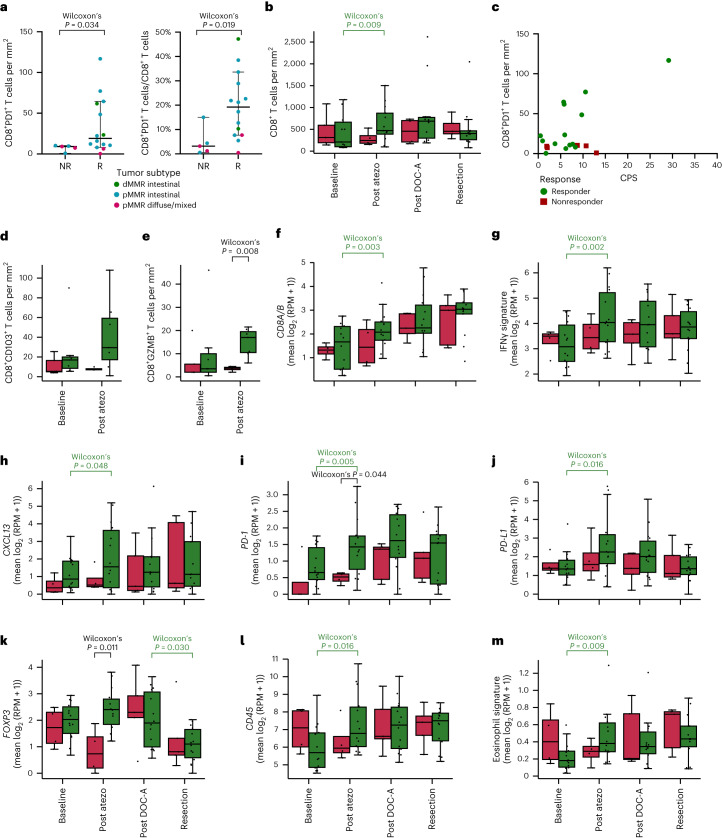
IHC for CD8^+^PD-1^+^ TCI plus CD8^+^ TCI and dynamics of immune-related gene expression in predicting response to ICB plus chemotherapy. **a**, Pretreatment CD8^+^PD-1^+^ T cells using IHC per mm^2^ (left) and as percentage of all CD8^+^ cells (right) in nonresponders (NR, *n* = 5) versus responders (R, *n* = 14). Dots represent individual patients. The horizontal line represents the median; whiskers show the 95% CIs. The difference between R and NR was tested using a two-sided Wilcoxon rank-sum test. **b**, Dynamics of CD8^+^ T cells per mm^2^ in R (green) versus NR (red) at baseline (R, *n* = 13; NR, *n* = 5) after monotherapy atezolizumab (post atezo; R, *n* = 13; NR, *n* = 4), after DOC plus atezolizumab (post DOC-A; R, *n* = 10; NR, *n* = 4) and at resection (R, *n* = 14; NR, *n* = 5). **c**, Scatter plot showing the relation between pretreatment PD-L1 CPS and CD8^+^PD-1^+^ T cells per mm^2^ in R (*n* = 14) versus NR (*n* = 5). Dots represent individual patients. **d**,**e**, Dynamics of CD8^+^CD103^+^ T cells per mm^2^ (**d**) and CD8^+^GZMB^+^ T cells per mm^2^ (**e**) analyzed by IMC in pMMR-complete responders (green) versus pMMR nonresponders (red) at baseline (R, *n* = 7; NR, *n* = 5) and after monotherapy atezolizumab (post atezo; R, *n* = 7; NR, *n* = 4). **f**–**m**, Dynamics of gene expression of *CD8* (=*CD8A* + *CD8B*) (**f**). IFNγ signature (**g**). *CXCL13* (**h**). *PD-1* (**i**). *PD-L1* (**j**). *FOXP3* (**k**). *CD45* (**l**). Eosinophil signature (**m**). **f**–**m**, Dynamics of gene expression in R (green) and NR (red) at baseline (R, *n* = 14; NR, *n* = 4) after monotherapy atezolizumab (post atezo; R, *n* = 14; NR, *n* = 4), after DOC plus atezolizumab (post DOC-A; R, *n* = 14; NR, *n* = 5) and at resection (R, *n* = 14; NR, *n* = 5). **b,d**–**m**, Box plots represent the median, 25th and 75th percentiles; whiskers extend from the hinge to the largest value below 1.5 × IQR from the hinge. Differences between R and NR were tested using a two-sided Wilcoxon rank-sum test. Differences between time points in R and NR separately were tested using a two-sided Wilcoxon signed-rank test. Only significant *P* values are shown; colors indicate a significant increase or decrease in responders (green) or a significant difference between responders and nonresponders (black). RPM, reads per million.

Expression of PD-1 on CD8^+^ tumor-infiltrating lymphocytes has previously been shown to accurately identify clonally expanded tumor-reactive T cells, indicating its potential as a predictive biomarker of antitumor responses induced by ICB^32^, and recent findings have provided further support for the predictive value of CD8^+^PD-1^+^ TCI^17,33,34^. In our study, CD8^+^PD-1^+^ TCI using IHC (Methods) demonstrated a significantly higher value at baseline in responders than in nonresponders (*P* = 0.034, Fig. 3a). Furthermore, the proportion of CD8^+^PD-1^+^ T cells among total CD8^+^ T cell numbers was significantly higher in responders than nonresponders (*P* = 0.019, Fig. 3a). Moreover, imaging mass cytometry (IMC) showed a higher baseline abundance of CD103^+^ CD8^+^ T cells in responders than nonresponders (Fig. 3d). Both PD-1 and CD103 are considered surrogates of presumed tumor-reactive T cells^34,35^. In contrast, no difference was observed in total CD8^+^ TCI between responders and nonresponders (Fig. 3b), adding to the evidence that the functional status of CD8^+^ T cells forms a critical parameter. In addition to CD8^+^PD-1^+^ TCI predicting ICB responsiveness, studies in anti-PD-1-treated non-small cell lung cancer (NSCLC) patients found a strong correlation between high CD8^+^PD-1^+^ TCI and durable treatment response as well as OS^33,34^. Along these lines, we also observed an association between the proportion of CD8^+^PD-1^+^ TCI and DFS, albeit not significant (*P* = 0.060), and OS (*P* = 0.099).

Importantly, and in contrast to findings in metastatic disease, where efficacy of immunotherapy is primarily observed in PD-L1 CPS-positive tumors, CPS was not predictive of response at cut-offs of 1, 5 or 10 (Supplementary Table 2). Moreover, four of 14 responders showed a CPS ≤ 5, whereas two of five nonresponders showed a CPS ≥ 10. Interestingly, tumors from nonresponders with a high CPS displayed relatively low CD8^+^PD-1^+^ TCI (Fig. 3c), again indicating the importance of the baseline presence of tumor-reactive T cells.

**Fig. 4:**
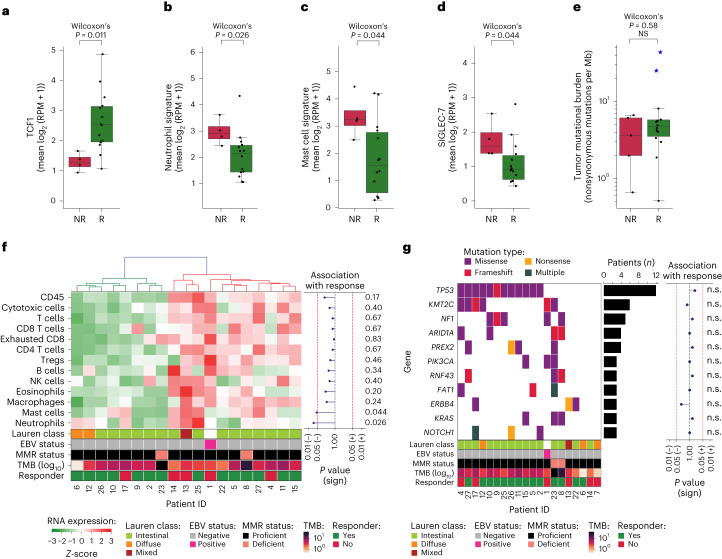
Comparisons between responders and nonresponders of pretreatment *TCF1* and *SIGLEC-7* gene expression, neutrophil and mast cell signatures, TMB, infiltration of immune cell subsets and driver gene mutations. **a**–**d**, Pretreatment gene expression in nonresponders (NR, *n* = 5) versus responders (R, *n* = 14) of *TCF1* (**a**), neutrophil signature (**b**), mast cell signature (**c**) and *SIGLEC-7* (**d**). Boxplots represent the median, 25th and 75th percentiles; whiskers extend from the hinge to the largest value below 1.5 * IQR from the hinge. The difference between NR and R was tested using a two-sided Wilcoxon Rank-sum test. **e**, TMB (number of nonsynonymous mutations per megabase of protein coding genome, *y* axis, log_10_scale) in nonresponders (NR, *n* = 5) versus responders (R, *n* = 14). Blue stars indicate patients with dMMR tumors. Box plots represent the median, 25th and 75th percentiles; whiskers extend from the hinge to the largest value below 1.5 × IQR from the hinge. The difference between NR and R was tested using a two-sided Wilcoxon rank-sum test. **f**, Heatmap of baseline RNA expression (*Z*-scores) of TME-specific signatures for leukocytes (CD45), cytotoxic cells, T cells, CD8 T cells, exhausted CD8 T cells, CD4 T cells, T_reg_ cells, B cells, NK cells, eosinophils, macrophages, mast cells and neutrophils. Patients (*x* axis) were ordered on the basis of hierarchical clustering (upper dendrogram). The squares below indicate Lauren classification, EBV status, MMR status, TMB (log_10_scale) and pathologic response per patient. The association of signature expression with response is shown on the right as a lollipop plot of signed, two-sided Wilcoxon rank-sum test-based *P* values (unadjusted for multiple hypothesis testing; *x* axis on log_10_ scale). **g**, Mutational status of all cancer-driver genes altered in ≥3 patients (colors denote the mutation type), alteration frequency (bar plot) and association with response (lollipop plot of signed, two-sided Fisher’s exact test-based *P* values (unadjusted for multiple hypothesis testing; *x*axis on log_10_ scale). The squares below indicate Lauren classification, EBV status, MMR status, TMB (log_10_ scale) and responder status per patient. n.s., not significant.

In addition, we performed transcriptional analysis of previously proposed biomarkers of response to ICB, including IFNγ signature^36^, *CD8A/B*, *CD274* (encoding PD-L1), *FOXP3* and *CXCL13*, encoding a chemokine that is associated with follicular helper T cells and involved in the formation of tertiary lymphoid structures^37^. These analyses did not show significant baseline differences between responders and nonresponders (Fig. 3f–m). Given the emerging evidence for TCF1 (encoded by *TCF7*) as a potential predictive biomarker of ICB response^38,39^, we assessed and found significantly higher baseline TCF1 RNA expression in responders than nonresponders (*P* = 0.011, Fig. 4a). TCF1 plays a principal role in T cell development, as it is a crucial component for differentiation of CD4^+^ T cells into T follicular helper cells as well as an identifying marker of stem-like CD8^+^ T cells with self-renewal capacity^39^.

To better understand possible underlying factors of nonresponse, we explored the presence of immunosuppressive features in the TME and found that baseline neutrophil signature was significantly higher in nonresponders than responders (*P* = 0.026, Fig. 4b). Considering that neutrophils, but also tumor-associated macrophages and myeloid-derived suppressor cells, may be recruited by mast cells^40^, we assessed and correspondingly found a significantly higher mast cell signature in nonresponders than responders (*P* = 0.044, Fig. 4c). Deconvolution of RNA sequencing data indicated no other significant associations between pathologic response and relative cell type compositions (Fig. 4f). In addition, expression of *SIGLEC-7*, an inhibitory checkpoint found on natural killer (NK) cells, T cells and dendritic cells and shown to promote immune suppression when bound to sialic acids on cancer cells, was significantly higher in nonresponders than responders (*P* = 0.044, Fig. 4d).

Genomic analyses showed that the genetic makeup of our cohort was representative of this patient population (Fig. 4g)^41^, increasing the likelihood that our findings will translate to the general patient population. Furthermore, these analyses did not show any significant associations between pathologic response and alterations of driver genes (Fig. 4g) or mutational signatures (Supplementary Fig. 2). Notably, pathologic responses were observed despite a low pretreatment tumor mutational burden (TMB), and TMB was not significantly different between responders and nonresponders, with a median of 4.71 (range 0.51–43.62) and 3.63 (range 0.66–6.66) mutations per megabase, respectively (*P* = 0.58, Fig. 4e).

A post hoc translational analysis excluding patients with dMMR tumors was performed, showing that baseline differences observed by transcriptomics and CD8^+^PD-1^+^ IHC were also present when considering only pMMR responders versus nonresponders (Supplementary Fig. 3).

### Atezolizumab leads to immune activation in the TME

A challenge in clinical immune-oncology studies that evaluate combination therapies has been to assign treatment-induced alterations of the TME to individual drugs or their combination. To understand whether therapy-induced TME alterations induced by the atezolizumab-chemotherapy combination are substantially different from the effects of atezolizumab monotherapy, we compared changes in the TME after the initial cycle of atezolizumab monotherapy to those observed on subsequent combination therapy.

Using IHC, a significant increase in CD8^+^ TCI in responding patients was observed after atezolizumab monotherapy (*P* = 0.009), whereas CD8^+^ TCI was stable in nonresponders (*P* = 1.0, Fig. 3b). Notably, the subsequent combination of atezolizumab with chemotherapy did not result in a further substantial increase in the CD8^+^ TCI in responders. In line with these data, analysis of transcriptomic data showed significantly increased *CD8A/B* expression after atezolizumab monotherapy solely in responders (*P* = 0.003), without a further significant change following the first combination cycle (Fig. 3f). IMC was performed to further characterize tumor-infiltrating immune cells in a subset of pMMR tumors, including nonresponders and responders with a pCR (Supplementary Fig. 4a). On monotherapy atezolizumab, a substantial increase in the expression of granzyme B in CD8^+^ T cells, indicating activation of this population, was observed specifically in responding patients (Fig. 3e). Because there is increasing evidence that the spatial proximity of immune cells to tumor cells may be predictive for response to ICB^42^, IMC data were also used to explore the spatial distribution of cells in the TME. A neighborhood analysis showed an enrichment of interactions between CD8^+^ T cells and cancer cells following monotherapy atezolizumab in responders (Supplementary Fig. 4b). Increased immune infiltration was also evident from transcriptomic data, showing increases in *CD45* and eosinophil signature expression (Fig. 3l,m). Furthermore, increased T cell activity on atezolizumab monotherapy was indicated by upregulation of an IFNγ signature^36^ and *PD-1*, *PD-L1* and *CXCL13* expression (Fig. 3g–j), whereas no further increase was observed after combination therapy. Notably, IMC demonstrated a higher proportion of HLA-DR^+^ cancer cells in responders than nonresponders after monotherapy atezolizumab (Supplementary Fig. 4c), which is in line with the increased IFNγ signaling in responders^43^. In line with this observed immune activation, *CD4* expression as well as expression of various inhibitory immune checkpoints, including *LAG3*, *SIGLEC-7* and *SIGLEC-9*, increased significantly in responders on atezolizumab monotherapy (Supplementary Fig. 5). After exclusion of dMMR tumors, the observed higher immune activation in responders after monotherapy atezolizumab generally held true, although to a slightly lesser extent (Supplementary Fig. 6).

Finally, building on recent data showing an important role for eosinophils in the response to ICB in NSCLC, breast and colon cancer^44^, on top of histopathologic findings of eosinophil infiltration after treatment in the current study, transcriptomic analysis showed a significant increase in eosinophil signatures in responders after atezolizumab monotherapy (*P* = 0.009), whereas a trend towards decrease was observed in nonresponders (*P* = 0.273, Fig. 3m). By analysis of delta (*Δ*) expression values to compare the changes between time points (Methods) in responders and nonresponders, we found the changes in *CD45*, eosinophils and *LAG3* after atezolizumab monotherapy to be significantly different between responders and nonresponders (Supplementary Fig. 7), implying that the observed differences are not driven solely by the relatively small number of nonresponders.

With the availability of biopsies at different time points per patient, we were also able to assess the dynamics of immune cell subsets after the subsequent addition of chemotherapy to atezolizumab. We found a substantial increase in CD45^+^ cells, CD4^+^ T cells and FOXP3^+^ regulatory T (T_reg_) cells in nonresponders, whereas these subsets decreased in responders (Fig. 3k,l and Supplementary Fig. 5), resulting in a significant difference between responders and nonresponders by comparison of *Δ* values (Supplementary Fig. 7).

As a control, transcriptomic analysis of samples at surgery showed that expression levels of above-mentioned immune-related genes remained relatively stable, with no significant changes relative to the samples obtained after the first combination cycle in either responders or nonresponders (Fig. 3 and Supplementary Fig. 5), emphasizing that the specific changes observed after atezolizumab were treatment-related.

Overall, these findings demonstrate, also on the basis of analysis of the TME, that the addition of atezolizumab has a major impact on the effect of chemotherapy in G/GEJ cancers.

## Discussion

Here we show that the combination of neoadjuvant atezolizumab plus chemotherapy is safe and has promising antitumor activity in G/GEJ adenocarcinoma, leading to an MPR in 70% and a pCR in 45% of patients. Importantly, pathologic response showed an excellent correlation with survival, with 13 of 14 responders without disease recurrence after a median follow-up of 47 months, whereas five of six nonresponders had a recurrence and died of their disease. Although previous studies in G/GEJ cancer have shown an association between response to neoadjuvant chemotherapy or chemoradiotherapy and outcome^45,46^, our data indicate that the association may be stronger and more similar to patients receiving neoadjuvant immunotherapy for NSCLC, melanoma, colon and bladder cancer, where patients with MPR and pCR have a negligible risk of disease recurrence. In line with prior research in colorectal cancer and esophagogastric tumors^47,48^, we observed an association between ctDNA and recurrence risk. In addition, presurgical ctDNA status was associated with pathologic response, highlighting potential clinical utility.

In the current study, we observed a 3-year recurrence rate of 27%, whereas the expected recurrence rate for G/GEJ cancers following FLOT chemotherapy is about 50% at 3 years^3^. Despite the limitations of cross-trial comparisons and our small patient cohort, these findings warrant validation in larger cohorts. The use of anti-PD-1/PD-L1 in this patient population is also supported by recent results from the DANTE study, in which patients were randomized to perioperative FLOT with or without atezolizumab, showing an increase of pCR in patients who received atezolizumab compared to FLOT only (24% versus 15%, respectively). In the phase 3 MATTERHORN study, the pCR and near-CR rates were 19% and 27% in patients treated with perioperative FLOT plus anti-PD-1 versus 7% and 14% in FLOT-treated controls^49^. In the similar ICONIC study, patients received perioperative FLOT plus avelumab with MPR and pCR rates of 21% and 15%, respectively^50^. The study was closed early, however, because the target pCR of 25% was unlikely to be achieved.

When considering only intestinal-type tumors in the PANDA study, MPR and pCR rates were 80% and 60%, respectively. Albeit in a small cohort, these pathologic responses compare favorably to historical data in similar patient populations. In the FLOT4-AIO study assessing perioperative FLOT chemotherapy, a near-pCR rate of 42%, including 23% pCR, was noted among patients with intestinal-type tumors^4^. Likewise, a comparable pCR rate of 23% was observed in patients with GEJ and esophageal adenocarcinoma after neoadjuvant chemoradiotherapy in the CROSS study, also a standard-of-care treatment regimen for patients with GEJ cancers^51^. Altogether, our results indicate that adding atezolizumab to neoadjuvant chemotherapy may lead to a higher pathologic response rate than expected with chemotherapy alone. Although a higher pathologic response rate is also observed in similar studies of ICB plus chemotherapy^49,50,52–57^, the seemingly higher responses achieved in our study may be attributed to differences in study design including induction treatment with atezolizumab monotherapy and total neoadjuvant treatment instead of perioperative treatment.

Notably, the translational work associated with this study strongly supports the added value of atezolizumab in neoadjuvant regimens for G/GEJ cancers. Specifically, the observation that pretreatment CD8^+^PD-1^+^ TCI was predictive of response is consistent with immune pressure being a determinant of efficacy of this treatment, in line with a recent study in ICB-treated gastric cancer patients reporting a strong association between clinical response to anti-PD-1/PD-L1 and the proportion of CD8^+^PD-1^+^LAG3^−^ T cells in proximity of tumor cells^42^. Second, and most importantly, our study design included a cycle of monotherapy atezolizumab before combination with chemotherapy, providing the opportunity to specifically capture the contribution of anti-PD-L1 and the subsequent combination with chemotherapy. The observed substantial immune activation following atezolizumab monotherapy and, by comparison, limited changes on subsequent combination therapy, form direct evidence for the contribution of PD-1/PD-L1 axis blockade to the observed resculpting of the TME. The increases in CD8^+^ TCI and immune-related gene expression following atezolizumab monotherapy were most evident in responders, indicating that analysis of the TME following the initial cycle of ICB may provide valuable predictive insights about the efficacy of this treatment strategy in individual patients. Nevertheless, it should be noted that a single dose of chemotherapy before the introduction of anti-PD-1 in gastric cancer patients has led to similar signs of immune activation^58^. Therefore, although our findings are consistent with ICB potentially priming the TME, a randomized comparison is warranted to clarify these data. Furthermore, FOXP3 demonstrated a substantial increase in nonresponders following combination therapy, with a corresponding significant difference in *Δ* values in responders versus nonresponders. These findings indicate that after chemotherapy, an immunosuppressive TME with high T_reg_ cell infiltration potentially contributes to ICB treatment failure and thus provides a topic for future investigation that may help unravel mechanisms underlying nonresponse to ICB^59^.

These observations, together with the predictive power of CD8^+^PD-1^+^ T cells, form an independent layer of evidence, on top of the clinical data, that atezolizumab has an additive effect together with chemotherapy in eliciting responses in G/GEJ cancers.

Importantly from the perspective of safety and feasibility, the incidence of chemotherapy-related AEs in the PANDA study was comparable to those reported in the FLOT-4AIO study^4^. Grade 3 irAEs occurred in only 14% of patients and were manageable, with symptoms resolving completely. A notable difference in favor of the total neoadjuvant treatment in the PANDA study compared to standard-of-care perioperative regimens with FLOT is the ability to complete all cycles of treatment when given only in a neoadjuvant setting, whereas in previous studies less than 50% of patients completed all adjuvant cycles of FLOT chemotherapy^3,60^. In our study, all but one patient proceeded to surgery, with an R0 resection rate of 95% and a similar postoperative complication rate compared to previous studies and real-world data from the Netherlands^61,62^, making this a well-tolerated treatment regimen with no new safety signals compared to treatment without immunotherapy.

Limitations of our study include the small number of patients and the single-arm design, making this a proof-of-concept study. Regardless, immune activation on atezolizumab monotherapy was unambiguous and most prominent in responding patients. Whether, next to pretreatment CD8^+^PD-1^+^ levels, the magnitude of this immune activation could be used to distinguish responders from nonresponders will require analysis in a larger cohort. Furthermore, the observed high T_reg_ cell infiltration in nonresponders, together with emerging evidence that T_reg_ cell-targeted approaches may enhance neoadjuvant ICB efficacy, prompt further exploration in future studies^63^. In addition to next-generation anti-CTLA-4, which has been shown to improve T_reg_ cell depletion^64,65^, antibodies targeting CCR8, a chemokine receptor selectively expressed on immunosuppressive T_reg_ cells in the TME, are being developed and may provide new avenues^66,67^.

Overall, the promising high pathologic response rate and the excellent survival of responders after neoadjuvant atezolizumab plus chemotherapy in G/GEJ adenocarcinoma warrant validation in a randomized controlled study to draw definitive conclusions. Additionally, on the basis of the observation that atezolizumab monotherapy leads to prominent changes in the TME, the question of whether selective absence of chemotherapy during the first treatment cycle is causally related to the observed high response rate may be explored.

## Methods

### Patient population

Eligible patients were 18 years of age or older and had previously untreated, histologically confirmed gastric or GEJ adenocarcinoma that was deemed resectable and showed no signs of distant metastases. Patients had an Eastern Cooperative Oncology Group (ECOG) performance status of 0 or 1 and adequate hematologic and end-organ function. Key exclusion criteria were clinical or radiological signs of esophageal perforation, immunodeficiency or immunosuppressive treatment, active or history of autoimmune disease and/or history of malignancy within 3 years before screening. Full inclusion and exclusion criteria were as follows.

#### Inclusion criteria

Inclusion criteria were as follows:

- Signed informed consent
- Primary resectable, histologically confirmed gastric or GEJ adenocarcinoma
- ECOG performance status of 0 or 1
- Age 18 or older
- No signs of distant metastases
- Adequate hematologic and end-organ function, defined by the following laboratory test results, obtained within 14 days before initiation of study treatment:
  - Absolute neutrophil count ≥ 1.5 × 10^9^ per l (1,500 per µl) without granulocyte colony-stimulating factor support
  - Lymphocyte count ≥ 0.5 × 10^9^ per l (500 per µl)
  - Platelet count ≥ 100 × 10^9^ per l (100,000 per µl) without transfusion
  - Hemoglobin ≥ 5,6 mmol per l (patients may be transfused to meet this criterion)
  - Aspartate aminotransferase, alanine aminotransferase and alkaline phosphatase ≤ 2.5 × upper limit of normal (ULN)
  - Serum bilirubin ≤ 1.5 × ULN except for patients with known Gilbert disease: serum bilirubin level ≤ 3 × ULN
  - Serum creatinine ≤ 1.5 × ULN or Creatinine clearance ≥ 40 ml per min (calculated using the Cockcroft–Gault formula)
  - Serum albumin ≥ 25 g per l
  - For patients not receiving therapeutic anticoagulation: international normalized ratio or activated partial thromboplastin time ≤ 1.5 × ULN
- For patients receiving therapeutic anticoagulation: stable anticoagulant regimen
- CT scan of thorax and abdomen <4 weeks before registration. PET scan and endoscopic ultrasound are required for GEJ tumors and are optional for gastric cancers
- For diffuse-type gastric cancers, diagnostic laparoscopy should be performed and show no signs of peritoneal metastases
- Patients must be willing to undergo esophagogastroduodenoscopy and biopsies before start of treatment and during treatment at defined time points
- Women of childbearing potential (WOCBP) must have a negative serum or urine pregnancy test (minimum sensitivity 25 IU per l or equivalent units of human chorionic gonadotropin) within 14 days before the start of treatment
- Men who are sexually active with WOCBP must use any contraceptive method with a failure rate of less than 1% per year. Men receiving atezolizumab and who are sexually active with WOCBP will be instructed to adhere to contraception for a period of 31 weeks after the last dose of investigational product. Women who are not of childbearing potential (that is, who are postmenopausal or surgically sterile) as well as azoospermic men do not require contraception
- For WOCBP (postmenarcheal, has not reached a postmenopausal state (≥12 continuous months of amenorrhea with no identified cause other than menopause) and has not undergone surgical sterilization by removal of ovaries and/or uterus): agreement to remain abstinent (refrain from heterosexual intercourse) or use contraceptive methods with a failure rate of <1% per year during the treatment period and for 5 months after the last dose of atezolizumab (bilateral tubal ligation, male sterilization, hormonal contraceptives that inhibit ovulation, hormone-releasing intrauterine devices and copper intrauterine devices).

#### Exclusion criteria

Exclusion criteria were as follows:

- Clinical symptoms or radiological suspicion of perforation
- Prior treatment for disease under study
- Signs or suspicion of metastatic disease
- Active or history of autoimmune disease or immune deficiency, including, but not limited to, myasthenia gravis, myositis, autoimmune hepatitis, systemic lupus erythematosus, rheumatoid arthritis, inflammatory bowel disease, antiphospholipid antibody syndrome, Wegener granulomatosis, Sjögren syndrome, Guillain–Barré syndrome or multiple sclerosis. Patients with a history of autoimmune-related hypothyroidism who are on thyroid-replacement hormone are eligible for the study. Patients with controlled Type 1 diabetes mellitus who are on an insulin regimen are eligible for the study. Patients with eczema, psoriasis, lichen simplex chronicus or vitiligo with dermatologic manifestations only (for example, patients with psoriatic arthritis are excluded) are eligible for the study provided all of the following conditions are met:
  - Rash must cover <10% of body surface area
  - Disease is well controlled at baseline and requires only low-potency topical corticosteroids
  - No occurrence of acute exacerbations of the underlying condition requiring psoralen plus ultraviolet A radiation, methotrexate, retinoids, biologic agents, oral calcineurin inhibitors or high-potency or oral corticosteroids in the previous 12 months
- History of idiopathic pulmonary fibrosis, organizing pneumonia (for example, bronchiolitis obliterans), drug-induced pneumonitis or idiopathic pneumonitis or evidence of active pneumonitis on screening chest CT scan. History of radiation pneumonitis in the radiation field (fibrosis) is permitted
- Known active tuberculosis
- Significant cardiovascular disease (such as New York Heart Association Class II or greater cardiac disease, myocardial infarction or cerebrovascular accident) within 3 months before initiation of study treatment, unstable arrhythmia or unstable angina
- Major surgical procedure other than diagnostic laparoscopy within 4 weeks before initiation of study treatment or anticipation of need for a major surgical procedure, other than for this diagnosis, during the study
- Severe infection within 4 weeks before initiation of study treatment, including, but not limited to, hospitalization for complications of infection, bacteremia or severe pneumonia
- Prior allogeneic stem cell or solid organ transplantation
- Any other disease, metabolic dysfunction, physical examination finding or clinical laboratory finding that contraindicates the use of an investigational drug, may affect the interpretation of the results or may render the patient at high risk from treatment complications
- Treatment with a live, attenuated vaccine within 4 weeks before initiation of study treatment or anticipation of need for such a vaccine during atezolizumab treatment or within 5 months after the last dose of atezolizumab
- Current treatment with anti-viral therapy for the hepatitis B virus
- Treatment with investigational therapy within 28 days before initiation of study treatment
- Prior treatment with CD137 agonists or ICB therapies, including anti-CTLA-4, anti-PD-1 and anti-PD-L1 therapeutic antibodies
- Treatment with systemic immunostimulatory agents (including, but not limited to, interferon and interleukin 2 (IL-2)) within 4 weeks or 5 half-lives of the drug (whichever is longer) before initiation of study treatment
- Conditions requiring systemic treatment with either corticosteroids (>10 mg daily prednisone equivalents) or other immunosuppressive medications within 14 days of study drug administration. Inhaled or topical steroids and adrenal replacement doses >10 mg daily prednisone equivalents are permitted in the absence of active autoimmune disease
- History of severe allergic anaphylactic reactions to chimeric or humanized antibodies or fusion proteins.
- Known hypersensitivity to Chinese hamster ovary cell products or to any component of the atezolizumab formulation
- Intercurrent illnesses, including, but not limited to, infections, that are determined incompatible with the study treatment and protocol by the study team
- Underlying medical conditions that will make the administration of the study drug hazardous or obscure the interpretation of toxicity determination of AEs
- Positive test for hepatitis B surface antigen or hepatitis C virus ribonucleic acid (HCV antibody) indicating acute or chronic infection
- History of testing positive human immunodeficiency virus or known acquired immunodeficiency syndrome (AIDS)
- History of uncontrolled medical or psychiatric illness.
- Psychological, familial, sociological or geographical condition potentially hampering compliance with the study protocol and follow-up schedule
- Pregnancy or breastfeeding or intention of becoming pregnant during study treatment or within months after the last dose of study treatment
- History of malignancy within 3 years before screening, with the exception of malignancies with a negligible risk of metastasis or death (for example, 5-year OS rate >90%), such as adequately treated carcinoma in situ of the cervix, nonmelanoma skin carcinoma, localized prostate cancer, ductal carcinoma in situ or Stage I uterine cancer

### Study design

The PANDA study (Clinicaltrials.gov: NCT03448835; EudraCT number: 2017-003854-17) is a single-arm, open-label, phase 2 study that was carried out at the Netherlands Cancer Institute. Because of the exploratory nature of this study, no formal sample size calculation was performed. The present study aimed to include a total of 20 patients (Extended Data Fig. 1). Sex and/or gender were not considered in the study design.

Patients were consulted in the outpatient clinic of the Netherlands Cancer Institute. Long-term follow-up was performed either at the outpatient clinic, by telephone or through telemedicine. Patients were treated with one cycle of atezolizumab 1,200 mg monotherapy on day 1. At 3 weeks (+/−2 days) and with intervals of 3 weeks between each cycle (weeks 3, 6, 9 and 12), patients received a total of four combination cycles consisting of atezolizumab 1,200 mg, docetaxel 50 mg m^−^^2^ and oxaliplatin 100 mg m^−^^2^ intravenously at the beginning of each cycle, plus oral capecitabine 850 mg m^−^^2^ twice daily on days 1–14 (DOC-A) of each cycle. Before docetaxel, patients received dexamethasone 8 mg orally on the day before and 4 mg intravenously on the day of infusion. To avoid high doses of steroids, which may affect ICB efficacy, the premedication dose of dexamethasone was lowered in comparison to the usually prescribed 16 mg for 3 days. All treatment cycles were given preoperatively, and patients received no standard adjuvant treatment. Surgery was scheduled 6–9 weeks after the start of the last treatment cycle. The type of surgical procedure was determined predominantly on the basis of tumor location. Patients underwent a transhiatal esophagectomy with gastric tube reconstruction and cervical anastomosis or either subtotal gastrectomy with Billroth II reconstruction or total gastrectomy with Roux-and-Y reconstruction. In all patients, a formal lymphadenectomy was performed. The surgical approach was open or minimally invasive.

### Endpoints and statistics

Primary endpoints were safety and feasibility. Safety analyses were performed in the PP population defined as all patients who received at least one dose of the study drugs (atezolizumab, docetaxel, oxaliplatin and capecitabine). Patients were monitored for (S)AEs until 100 days after the last dose of study treatment, and events were scored according to the National Cancer Institute Common Terminology Criteria for Adverse Events, v.4.03 (ref. ^69^). The most severe toxicity grade over all cycles according to the National Cancer Institute Common Terminology Criteria for Adverse Events v.4.03 was depicted per body system. Safety was assessed by the occurrence of AEs, serious AEs and treatment-related complications leading to delays in systemic treatment and/or surgery past 9 weeks after start of the last treatment cycle. Anastomotic leakage was defined as (1) clinical signs of a salivary fistula and/or (2) endoscopic evidence of an anastomotic defect. Feasibility was determined by adherence to the timeline according to the study protocol. Secondary and translational endpoints were analyzed in the PP population and included DFS calculated from the date of surgery, OS calculated from the date of enrollment, radiologic tumor regression assessed before cycle 4 of combination treatment, efficacy evaluated by histopathological response to treatment and associations between pathologic response and genomics, transcriptomics, IHC and IMC findings and ctDNA, including the TMB, gene expression signatures and TCI. Radiologic response assessment on CT was performed according to RECIST v.1.1. In absence of measurable lesions, considering that the primary tumor in a hollow organ cannot be assessed according to RECIST 1.1, response assessment was provided in a descriptive manner. FDG-PET images were analyzed for SUV_max_, SUV_mean_, SUV_peak_, MTV (calculated as volume with ≥50% SUV_max_) and TLG (calculated by multiplying MTV with SUV_mean_). Patients who underwent an evaluation scan were assigned to one of the following categories: complete metabolic response (FDG-uptake indistinguishable from surrounding background and less than liver), near-complete response (FDG-uptake similar or less than liver but still distinguishable from the rest of the stomach because of prior knowledge of tumor location, therefore possibly posttreatment inflammation rather than residual tumor), partial response (>30% decrease in FDG-uptake compared to baseline), nonresponse (FDG-uptake ≤30% of baseline) and progression (>30% increase in FDG-uptake, size or new lesion).

Before the start of treatment, clinical stage was assessed by physical examination, esophagoduodenoscopy with representative biopsies from tumor and normal tissue, blood tests and CT scan of thorax and abdomen. For GEJ tumors, endoscopic ultrasound and fluorine-18-deoxyglucose positron emission tomography (FDG-PET) CT were also performed according to local guidelines. For cT3 and cT4 gastric cancers, diagnostic laparoscopy was performed to exclude signs of peritoneal metastases according to national guidelines. Baseline clinical staging of primary tumors and lymph nodes was done according to the 8^th^ edition of the *AJCC Cancer Staging Manual*^68^. Radiographic restaging was performed before the last cycle of treatment by means of CT and/or FDG-PET CT. Blood samples, including peripheral blood mononuclear cells (PBMCs), were obtained before each treatment cycle, before surgery and during follow-up. On-treatment endoscopy with biopsies was performed after the initial cycle of atezolizumab monotherapy and again after the first cycle of DOC-A combination treatment. Posttreatment tissue was obtained by surgical resection. All tissue samples were directly frozen or formalin-fixed and embedded in paraffin.

Baseline characteristics are presented for the intention-to-treat population defined as all patients enrolled in the study. Categorical variables are summarized as absolute numbers and percentages and continuous variables with medians and (interquartile) ranges. For comparison of changes between subsequent time points, *Δ* expression values were calculated by subtracting the RNA expression value at a time point from the expression value at the subsequent time point. For analyses of continuous biomarker variables (except differential gene expression analysis; see below), differences between groups, including responders versus nonresponders, were analyzed using the Wilcoxon’s rank-sum test (Mann–Whitney *U-*test), whereas differences between pre-, on- and posttreatment measurements in a group were analyzed using Wilcoxon’s signed-rank test. Categorical variables were compared between groups using Fisher’s exact test. For binary outcomes, exact two-sided 95% CIs were calculated using the Clopper–Pearson method. The Kaplan–Meier method was used to analyze time-to-event endpoints. A log-rank test was used to compare DFS and OS curves between responders and nonresponders; for comparison of the OS curves, landmark analysis was performed with a landmark at the date of surgery. A Cox survival model was used to test the impact of CD8^+^PD-1^+^ TCI on survival. Median follow-up time from enrollment was calculated using the reverse Kaplan–Meier method. All reported *P* values are two-sided, and a *P* value of <0.05 was considered statistically significant. Analyses were performed using R v.4.2.2 (ref. ^70^), with the exception of genomic and transcriptomic biomarker analyses, which were performed in Python v.3.7.4 with Jupyter Notebook v.6.0.1 (using the following packages: Pandas v.0.25.1 for data-frame operations, Scipy v.1.3.1 for statistical testing, Decimal v.1.70 for decimal operations and Matplotlib v.3.2.1 plus Seaborn v.0.9.0 for visualization). Experiments performed on patient-derived material were not repeated.

### Study oversight

This study was conducted in accordance with the International Conference on Harmonization *Guideline for Good Clinical Practice* and the Declaration of Helsinki. The study protocol was approved by the Institutional Review Board of the NKI. All patients provided written informed consent before enrollment. There was no Data Safety Monitoring Board.

### Pathology assessments and IHC analyses

Formalin-fixed, paraffin-embedded (FFPE) sections were obtained from biopsies taken before and during treatment as well as from resection specimens. MMR status was determined in baseline tumor biopsies by IHC for MLH1, PMS2, MSH2 and MSH6 performed on a BenchMark Ultra autostainer (Ventana Medical Systems) according to manufacturer instructions. Briefly, paraffin sections were cut in 3 µm slides, heated at 75 °C for 28 minutes and deparaffinized with EZ Prep solution (Ventana Medical Systems). Heat-induced antigen retrieval was carried out using Cell Conditioning Solution 1 (CC1, Ventana Medical Systems) for 4 minutes at 95 °C. MLH1 was detected with Ready-to-Use M1 (6472966001, Roche); PMS2, 1:40 dilution, clone EP51 (M3647, Agilent Technologies); MSH2, Ready-to-Use G219-1129 (5269270001, Roche); MSH6, 1:50 dilution, EP49 (AC-0047, Abcam). Bound antibody was detected using the OptiView DAB Detection Kit, and slides were counterstained with Hematoxylin and Bluing Reagent (Ventana Medical Systems). Staining for MLH1, PMS2, MSH2 and MSH6 was scored as present/positive if convincing nuclear staining in tumor cells with a positive internal control was observed. The tumors were considered MMR deficient if at least one of the stains was absent/negative. HER2 status was determined during routine diagnostic workup using initial IHC testing, followed by ISH when the IHC result was 2+ (equivocal), according to the guidelines form the College of American Pathologists, American Society for Clinical Pathology and American Society of Clinical Oncology^71^.

Two experienced gastrointestinal pathologists performed histopathologic examination of biopsies and resection specimens. Slides were counterstained with Hematoxylin and Bluing Reagent (Ventana Medical Systems). The entire resected tumor and all lymph nodes were evaluated, and histopathologic tumor regression was assessed by estimating the percentage of RVT in the macroscopically identifiable tumor bed, as identified on routine hematoxylin and eosin (H&E) staining^27^. In addition, regression was classified according to the Mandard tumor regression grade (TRG)^72^: TRG1 (no residual tumor cells), TRG2 (rare residual tumor cells, near-pCR), TRG3 (fibrosis outgrowing residual tumor), TRG4 (residual tumor outgrowing fibrosis), TRG5 (no tumor regression). In line with recommendations for pathologic assessment of NSCLC and melanoma surgical specimens after neoadjuvant immunotherapy, MPR was defined as ≤10% RVT, corresponding to Mandard TRG1 (CR) or 2 (near-CR)^73^. Pathologic complete response (pCR) was equivalent to Mandard TRG1 and was defined as 0% RVT in both the primary tumor and lymph nodes. Partial response (PR) was defined as 50% or less RVT. When analyzing responders versus nonresponders, patients with an MPR (≤10% RVT) equivalent to Mandard TRG1,2 were categorized as responders, and tumors with a non-MPR (>10% regression) equivalent to TRG3–5 were classified as nonresponders.

FFPE specimens were additionally assessed by IHC analysis of CD8, PD-L1 and PD-1. IHC analysis after single stain of CD8 and PD-L1 was performed on a BenchMark Ultra autostainer (Ventana Medical Systems). An IHC double stain using CD8 and PD-1 was performed on a Discovery Ultra autostainer. Briefly, paraffin sections were cut at 3 µm, heated at 75 °C for 28 minutes and deparaffinized in the instrument with EZ Prep solution (Ventana Medical Systems). Heat-induced antigen retrieval was carried out using Cell Conditioning Solution 1 (CC1, Ventana Medical Systems) for 32 minutes at 95 °C (CD8), 48 minutes at 95 °C (PD-L1) or 64 minutes at 95 °C. (PD-1/CD8 double).

CD8 was detected using clone C8/144B (1:100 dilution, 32 minutes at 37 °C, Agilent/DAKO, lot 41311427) and PD-L1, clone 22C3 (1:40 dilution, 1 h at room temperature, Agilent/DAKO, lot 11295663). Bound antibody was detected using the OptiView DAB Detection Kit (Ventana Medical Systems). Slides were counterstained with Hematoxylin and Bluing Reagent (Ventana Medical Systems).

For the double staining of PD-1 (yellow) followed by CD8 (purple), PD-1 was detected in the first sequence using clone CAL20 (1:250 dilution, 1 h at room temperature, Abcam, lot GR3361014-6). PD-1 bound antibody was visualized using Anti-Mouse NP (Ventana Medical systems) for 12 minutes at 37 °C followed by Anti-NP AP (Ventana Medical systems) for 12 minutes at 370 °C, followed by the Discovery Yellow detection kit (Ventana Medical Systems). In the second sequence of the double staining procedure, CD8 was detected using clone C8/144B (1:200 dilution, 32 minutes at 37 °C, Agilent). CD8 was visualized using Anti-Mouse HQ (Ventana Medical systems) for 12 minutes at 370 °C followed by Anti-HQ HRP (Ventana Medical systems) for 12 minutes at 37 °C, followed by the Discovery Purple Detection Kit (Ventana Medical Systems). Slides were counterstained with Hematoxylin and Bluing Reagent (Ventana Medical Systems).

A PANNORAMIC 1000 scanner from 3DHISTECH was used to scan the slides at a ×40 magnification. Digital imaging analysis was performed using HALO imaging analysis software v.3.4.2986.185 (Indica Labs). Tumor areas were manually annotated by an experienced pathologist. Results presented here were obtained by measuring the entire tumor area followed by quantification of stained cells in these areas. For the CD8^+^ single stain, quantification of CD8^+^ cells was performed using a custom trained Cellpose^74^ network in QuPath v.0.3.2 using QuPath-extension-cellpose v.0.5.1 (ref. ^75^). For the double stain, CD8^+^, PD-1^+^ and CD8^+^PD-1^+^ cells were quantified using HALO algorithms, and figures (Fig. 3a,c) depicting this analysis were generated using GraphPad Prism v.9.0.2.

### IMC

IMC was performed on biopsies obtained at baseline and after monotherapy atezolizumab from a subset of patients with pMMR tumors, including nonresponders and responders with a pCR. FFPE sections of 4-µm thickness were subjected to IMC and labeled with 40 antibodies against cellular targets as described previously^76^. In short, tissue sections were deparaffinized by consecutive incubations in Xylol and ethanol, followed by heat-mediated antigen retrieval in high-pH Antigen Retrieval Solution (eBioscience, Thermo Fisher Scientific). After allowing the slides to cool for 1 hour, the sections were blocked with SuperBlock solution (Thermo Fisher Scientific) for 30 minutes and incubated overnight at 4 °C with anti-CD4 and anti-TCR gamma delta (antibody details are available in Supplementary Table 3). The next day, antibodies were washed away with PSB (supplemented with 1% BSA and 0.05% Tween) and sections were incubated 1 hour with metal conjugated Anti-Mouse (ab6708, Abcam) and Anti-Rabbit (ab6702, Abcam) antibodies. Next, sections were incubated for 5 hours at room temperature with an antibody mix containing the first set of antibodies followed by overnight incubation at 4 °C with the second set of antibodies. All antibodies were previously tested for optimal incubation temperature. Finally, sections were incubated for 5 minutes with DNA Intercalator Iridium (1.25 µM, Fluidigm), washed with PBS and water, and air-dried.

For each section, depending on tissue size, one or two 1 mm^2^ regions of interest were ablated on the Hyperion mass cytometry imaging system (Fluidigm). Data quality was visually inspected using the Fluidigm MCD viewer (v.1.0.560.6) and exported as multi-tiff files. Images were normalized by rescaling all images and markers between 0 and 1 followed by a two-step denoising where first a minimal signal threshold of 0.1 was set followed by per-marker percentile normalization. Cell segmentation masks were generated from the normalized images using CellProfiler (v.4.2.1). First nuclei were defined using the DNA images to which membranes were added using keratin, vimentin and CD45 images. Single-cell marker expression flow cytometry standard files were generated by combining the normalized images with cell segmentation masks in ImaCytE^77^, and after dimensionality reduction, cells were clustered by mean-shift clustering in Cytosplore (v.2.3.1)^78^. Clusters were mapped back on the images and visually confirmed by comparison with raw images in the MCD viewer. Finally, cluster abundances per image were combined per sample and visualized as cells per mm^2^. Because of low abundance of CD103 and Granzyme B, no distinct clusters were formed, and thus their presence was determined by counting the number of cells with a marker expression above 0.2 in each T cell cluster. Spatial cell–cell interactions were defined as all cells localizing within a 10-µm distance of the cell of interest. To account for random localization of highly abundant cells, a 500-iteration permutation test was used in which all cells were randomly distributed throughout the image. All interactions with a *Z*-score greater than 2 were considered specific. Data were combined and visualized in RStudio (R v.4.2.0) using the ggplot2 (v.3.4.0) and ComplexHeatmap (v.2.14.0) packages.

### Genomic and transcriptomic analyses

Whole-exome sequencing was performed on pretreatment tumor samples and matched germline DNA from PBMCs. RNA was isolated from tumor samples taken pre-, on- and posttreatment to determine expression of various individual genes as well as immune-related gene signatures.

DNA and RNA were extracted from fresh-frozen samples obtained pre- and posttreatment. For isolation, 10-µm slides were cut in a cryostat. Of the 10-µm slides, one 5-µm slide was cut and stained with H&E to assess tumor percentage. Samples were selected for isolation on the basis of a tumor percentage of at least 10%, except for posttreatment samples from patients with an MPR or pCR. DNA and RNA were isolated simultaneously with the AllPrep DNA/RNA/miRNA Universal Isolation Kit (Qiagen, 80224) by using the QIAcube according to manufacturer’s protocol.

#### DNA sequencing

The total amount of DNA was quantified on the Nanodrop 2000 (Thermo Fisher), and the amount of double-stranded DNA in the genomic samples was quantified using the Qubit dsDNA HS Assay Kit (Invitrogen, cat. no. Q32851). A maximum amount of 2,000 ng double-stranded genomic DNA was fragmented by Covaris shearing to obtain fragment sizes of 200–300 bp. Samples were purified using 2× Agencourt AMPure XP PCR purification beads according to the manufacturer’s instructions (Beckman Coulter, cat. no. A63881). The sheared DNA samples were quantified and qualified on a BioAnalyzer system using the DNA7500 assay kit (Agilent Technologies, cat. no. 5067–1506). With a maximum input micrograms of sheared DNA, library preparation for Illumina sequencing was performed using the KAPA HTP Prep Kit (KAPA Biosystems, KK8234). During library amplification, four PCR cycles were performed to obtain enough yield for exome capture. After library preparation, the libraries were cleaned using 1× AMPure XP beads. All DNA libraries were analyzed on a BioAnalyzer system using the DNA7500 chips to determine the concentration. Four pools of ten samples were created using 500 ng of each indexed sample. The pool was captured with an xGen Exome V2.0 probe (IDT, cat. no. 10005152) following the IDT xGen capture (IDT, cat. no. 1080577). After capture, the pool was amplified by ten PCR cycles and purified with Ampure XP beads. The pool was diluted to a final concentration of 10 nM and then subjected to sequencing on an Illlumina Novaseq 6000 machine with an S1 flowcell (sequenced at paired-end 100 bp) according to the manufacturer’s instructions. After paired-end adapter trimming using SeqPurge (v.2019 09), samples were aligned to the human reference genome (GRCh38) with the BWA (v.0.7.17) MEM algorithm, and UmiAwareMarkDuplicatesWithMateCigar (Genome Analysis Toolkit pipeline, v.4.2) was used to indicate the duplicate status. BaseRecalibrator (Genome Analysis Toolkit pipeline, v.4.2.) was run to readjust qualities after alignment, taking into account common SNPs (dbSNP v.151). Somatic mutation calling was performed using Mutect2 with default settings; however, as Mutect2’s per-patient thresholds for mutation calling limits the interpatient comparability of the TMB, we set a uniform mutation calling threshold of TLOD > 7.5 to ensure consistency about the specificity of mutation calls across all samples. For each patient, the TMB was calculated on the basis of whole-exome sequencing as the total number of nonsynonymous somatic mutations divided by the number of protein coding megabases with at least 25× sequencing coverage for the sample. To characterize the mutational driver landscape of the cohort, we considered all nonsynonymous mutations affecting oncogenes and tumor suppressors in the cancer-driver catalog, as previously defined^79^. For each sample, the activity of Pan-Cancer Analysis of Whole Genomes mutational signatures^80^ was calculated with the R package mutSigExtractor, v.1.28 (https://github.com/UMCUGenetics/mutSigExtractor). Consistent with similar work in the field^58^, we considered interpretable mutational signatures associated with known biology: age (SBS1, SBS5), apolipoprotein B mRNA-editing enzyme, catalytic polypeptide activity (SBS2, SBS13), ultraviolet light (SBS7a, SBS7b, SBS7c, SBS7d), tobacco exposure (SBS4), homologous recombination deficiency (SBS3), dMMR (SBS6, SBS15, SBS20, SBS26), nucleotide excision repair deficiency (SBS8), DNA proofreading deficiency (SBS10a, SBS10b) and base excision repair deficiency (SBS18).

#### RNA sequencing

Quality and quantity of the total RNA was assessed by the 2100 Bioanalyzer using a Nano chip (Agilent). The percentage of RNA fragments >200 nt fragment distribution values (DV200) were determined using the region analysis method according to the manufacturer’s instructions (Illumina, technical note 470-2014-001). Strand-specific libraries were generated using the TruSeq RNA Exome Library Prep Kit (Illumina) according to the manufacturer’s instructions (Illumina, no. 1000000039582v01). Briefly, total RNA was random primed and reverse-transcribed using SuperScript II Reverse Transcriptase (Invitrogen, part no. 18064-014) with the addition of Actinomycin D. Strand synthesis was performed using Polymerase I and RNaseH, replacing deoxythymidine triphosphate with deoxyuridine triphosphate. The generated cDNA fragments were 3′ end adenylated and ligated to Illumina Paired-End sequencing adapters and subsequently amplified by 15 cycles of PCR. The libraries were validated on a 2100 Bioanalyzer using a 7500 chip (Agilent) followed by 1–4 plex library pooling containing up to 200 ng of each sample.

The pooled libraries were enriched for target regions using the probe Coding Exome Oligos set (CEX, 45 MB) according to the manufacture’s instructions (Illumina, no. 1000000039582v01). Briefly, cDNA libraries and biotin-labeled capture probes were combined and hybridized using a denaturation step of 95 °C for 10 minutes and an incubation step from 94 °C to 58 °C having a ramp of 18 cycles with 1 minute incubation and 2 °C per cycle. The hybridized target regions were captured using streptavidin magnetic beads and subjected to two stringency washes, an elution step and a second round of enrichment followed by cleanup using AMPure XP beads (Beckman, A63881) and PCR amplification of ten cycles. The target-enriched pools were analyzed on a 2100 Bioanalyzer using a 7500 chip (Agilent), diluted and subsequently pooled equimolar into a multiplex sequencing pool. The libraries were sequenced with 54 paired-end reads on a NovaSeq6000 using an S1 Reagent Kit v.1.5 (100 cycles) (Illumina). Adapter trimming of stranded, paired-end RNAseq reads was performed using SeqPurge (v.2019 09). Then, samples were aligned to the human reference genome (GRCh38) using hisat2 (v.2.1.0). Strand-specific counts per gene were calculated using gensum (https://github.com/NKI-GCF/gensum) and Ensembl gtf GRCh38.102.

The expression of specific marker gene sets was calculated as the mean log_2_(reads per million) expression of the genes in the gene set (Supplementary Table 4 and refs. ^36,44,81,82^). To compare temporal expression changes between response groups, we calculated patient-specific *Δ* expression values of genes and gene sets by calculating the increase in log_2_(reads per million) expression over time.

### Personalized circulating tumor DNA analysis

WES was performed on tumor tissue and matched normal DNA. After quality metrics and sample concordance checks, WES data were used for somatic variant calling using Natera’s proprietary bioinformatics pipeline^83^. This method allows for filtering out putative germline and clonal hematopoiesis of indeterminate potential mutations. A prioritized list of up to 16 somatic single-nucleotide variants were selected, for which PCR amplicons were designed and applied to cfDNA of all patients^83^.

A median of 22.5 ng cell-free DNA was extracted from a median of 3.6 ml (range 0.9–4.9 ml) of plasma. Following this, cfDNA libraries were prepared using up to 66 ng cfDNA and subjected to end-repairing, A-tailing and adapter ligation, followed by amplification and purification of the product. Following library preparation, a multiplex targeted PCR was conducted on an aliquot of the cfDNA library, followed by amplicon-based sequencing at an average depth of >100,000 on the Novaseq platform. To consider a plasma sample ctDNA-positive, a cutoff of ≥2 variants detected out of 16 was used as a criterion. The ctDNA concentration was measured and reported as mean tumor molecules per milliliter of plasma^83^.

### Statistical analysis for ctDNA

Fisher’s exact test was used to compare categorical variables. Survival analyses were carried out with R software v.4.2.2 using the survival, survminer and coxphf packages. The Kaplan–Meier method was used to estimate the survival distribution. Differences between groups were tested using the log-rank test. To account for immortal time bias, a landmark analysis was performed at 12 weeks after surgery, whereby DFS was measured starting from day 90. Analysis of ctDNA concentration between responders and nonresponders at a post-neoadjuvant time point was performed using the ggplot2 package v.3.3.6 in R v.4.2.2. *P* values < 0.05 were considered statistically significant. Box plots were generated using the ggplot2 package v.3.3.6 in R v.4.2.2.

### Reporting summary

Further information on research design is available in the Nature Portfolio Reporting Summary linked to this article.

## Online content

Any methods, additional references, Nature Portfolio reporting summaries, source data, extended data, supplementary information, acknowledgements, peer review information; details of author contributions and competing interests; and statements of data and code availability are available at 10.1038/s41591-023-02758-x.

### Supplementary information

Supplementary Information Supplementary Figs. 1–6 and Tables 1–4.

Reporting Summary

## Figures and Tables

**Extended Data Table 1:**
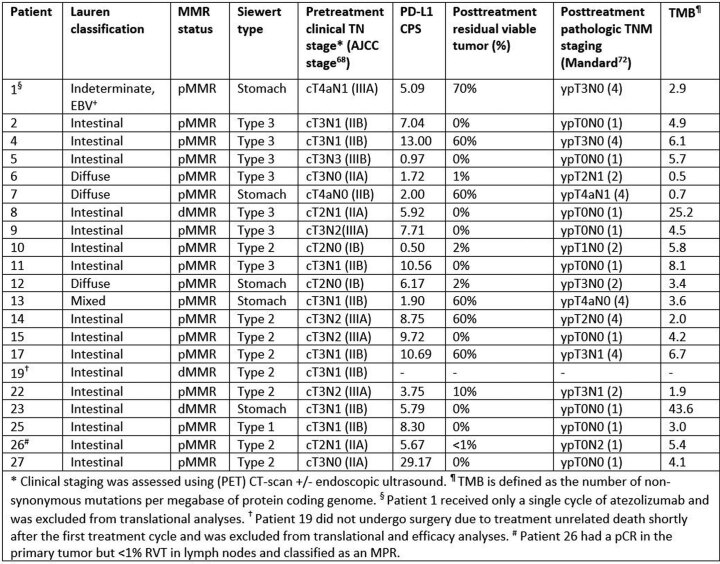
Clinical staging, histopathologic findings and tumor mutational burden (TMB) per patient

**Extended Data Table 2:**
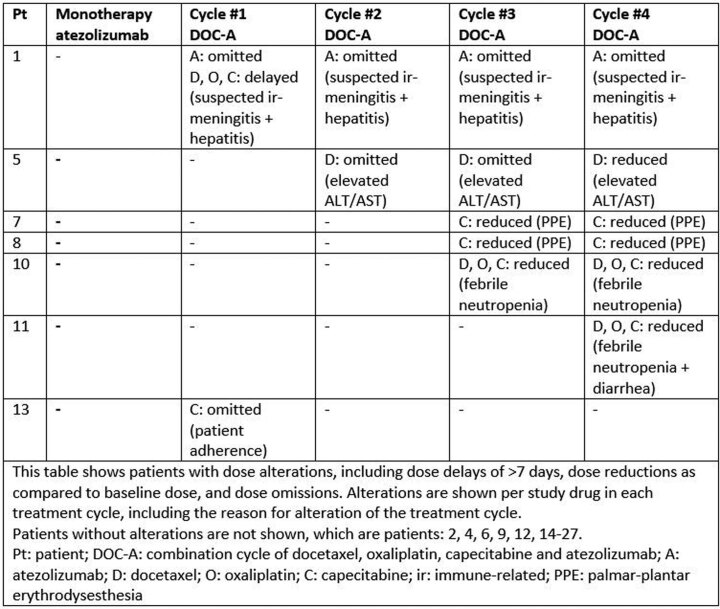
Dose delays, dose reductions and dose omissions per drug in each treatment cycle per patient in the per protocol population (*n* = 20)

**Extended Data Table 3:**
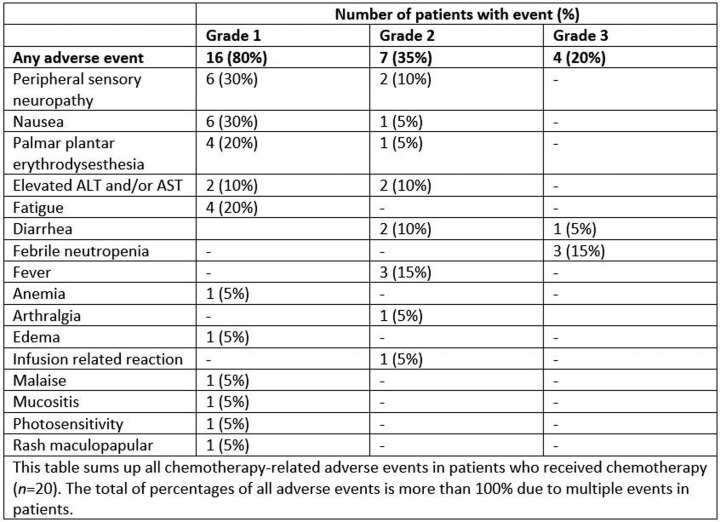
Chemotherapy-related adverse events (n = 20)

**Extended Data Table 4:**
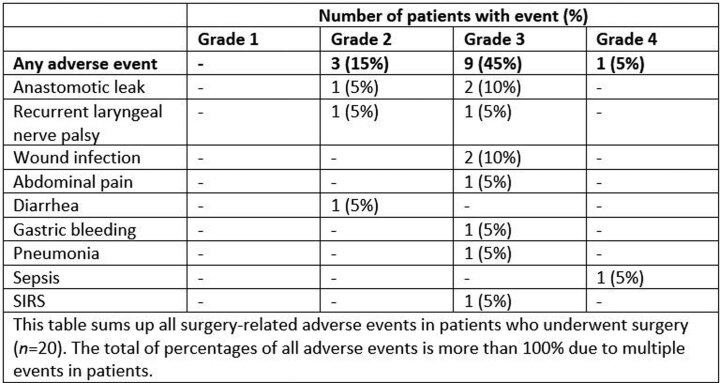
Surgery-related adverse events (n = 20)

**Extended Data Table 5:**
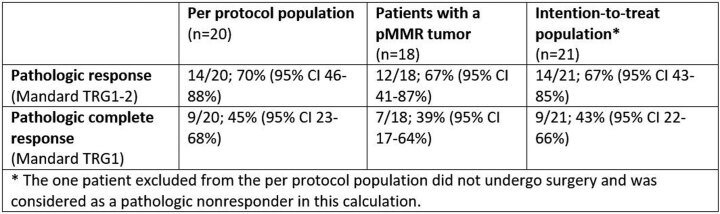
Pathologic response rate

## Data Availability

The RNA and DNA sequencing data will be deposited in the European Genome-Phenome Archive under EGAS50000000168, and these data will be shared on reasonable request for academic use and within the limitations of the provided informed consent and under General Data Protection Regulation law. All data requests supported by the principal investigator/corresponding author of the study will be reviewed by the institutional review board of the NKI. The researcher will need to sign a data access agreement with the NKI after approval. The form to request access can be found at https://ega.nki.nl/ and will be centrally reviewed through repository@nki.nl, which will contact the corresponding author (M.C.). Estimated time to response is 2–4 weeks.
