# Author Correction: Neoadjuvant atezolizumab plus chemotherapy in gastric and gastroesophageal junction adenocarcinoma: the phase 2 PANDA trial

**DOI:** 10.1038/s41591-024-02898-8

**Published:** 2024-03-06

**Authors:** Yara L. Verschoor, Joris van de Haar, José G. van den Berg, Johanna W. van Sandick, Liudmila L. Kodach, Jolanda M. van Dieren, Sara Balduzzi, Cecile Grootscholten, Marieke E. IJsselsteijn, Alexander A. F. A. Veenhof, Koen J. Hartemink, Marieke A. Vollebergh, Adham Jurdi, Shruti Sharma, Erik Spickard, Emilia C. Owers, Annemarieke Bartels-Rutten, Peggy den Hartog, Noel F. C. C. de Miranda, Monique E. van Leerdam, John B. A. G. Haanen, Ton N. Schumacher, Emile E. Voest, Myriam Chalabi

**Affiliations:** 1 Department of Gastrointestinal Oncology, Netherlands Cancer Institute – Antoni van Leeuwenhoek Hospital, Amsterdam, the Netherlands; 2 Department of Molecular Oncology and Immunology, Netherlands Cancer Institute – Antoni van Leeuwenhoek Hospital, Amsterdam, the Netherlands; 3 Oncode Institute, Amsterdam, the Netherlands; 4 Department of Pathology, Netherlands Cancer Institute – Antoni van Leeuwenhoek Hospital, Amsterdam, the Netherlands; 5 Department of Surgery, Netherlands Cancer Institute – Antoni van Leeuwenhoek Hospital, Amsterdam, the Netherlands; 6 Biometrics department, Netherlands Cancer Institute – Antoni van Leeuwenhoek Hospital, Amsterdam, the Netherlands; 7 Department of Pathology, Leiden University Medical Center, Leiden, the Netherlands; 8 Department of Medical Oncology, Netherlands Cancer Institute – Antoni van Leeuwenhoek Hospital, Amsterdam, the Netherlands; 9 Natera, Inc, Austin, TX USA; 10 Department of Nuclear Medicine, Netherlands Cancer Institute – Antoni van Leeuwenhoek Hospital, Amsterdam, the Netherlands; 11 Department of Radiology, Netherlands Cancer Institute – Antoni van Leeuwenhoek Hospital, Amsterdam, the Netherlands; 12 Department of Gastroenterology and Hepatology, Leiden University Medical Center, Leiden, the Netherlands; 13 Department of Medical Oncology, Leiden University Medical Center, Leiden, the Netherlands; 14 Oncology Service, Centre Hospitalier Universitaire Vaudois, Lausanne, Switzerland; 15 Department of Hematology, Leiden University Medical Center, Leiden, the Netherlands

**Keywords:** Phase II trials, Tumour immunology, Biomarkers, Gastric cancer

Correction to: *Nature Medicine* 10.1038/s41591-023-02758-x, published online 8 January 2024.

In the version of the article initially published, the Data Availability section and Reporting Summary contained the wrong accession code for the data deposited in the European Genome-Phenome Archive. This has now been updated to “EGAS50000000168” in the HTML and PDF versions of the article.

