# Supplementary material for: Neoadjuvant atezolizumab plus chemotherapy in gastric and gastroesophageal junction adenocarcinoma: the phase 2 PANDA trial
Source: Nat Med. 2024 Jan 8;30(2):519–30. doi: 10.1038/s41591-023-02758-x (PMC10878980; doi:10.1038/s41591-023-02758-x)
Supplement: Supplementary file 1 — Supplementary Figs. 1–6 and Tables 1–4. [file 41591_2023_2758_MOESM1_ESM.pdf]

nature medicine

Article

https://doi.org/10.1038/s41591-023-02758-x

# Neoadjuvant atezolizumab plus chemotherapy in gastric and gastroesophageal junction adenocarcinoma: the phase 2 PANDA trial

In the format provided by the authors and unedited

## Table of content

### SUPPLEMENTARY FIGURES

**Supplementary Figure 1**. Overview of samples used for translational analyses.
**Supplementary Figure 2**. Association of mutational signatures with response.
**Supplementary Figure 3**. Comparisons between pMMR responders and pMMR nonresponders of pretreatment TCF1 and SIGLEC-7 gene expression, neutrophil and mast cell signature plus immunohistochemistry for CD8+PD1+.
**Supplementary Figure 4**. Imaging mass cytometry of pMMR nonresponders and pMMR responders with a pCR.
**Supplementary Figure 5**. Dynamics of CD4 T-cells, LAG3, SIGLEC-7 and SIGLEC-9 and comparison between responders and nonresponders.
**Supplementary Figure 6**. Dynamics of immune-related gene expression in predicting response to ICB plus chemotherapy comparing pMMR responders and pMMR nonresponders
**Supplementary Figure 7**. Dynamics of immune-related gene expression presented as delta (Δ) values in responders and nonresponders.

### SUPPLEMENTARY TABLES

**Supplementary table 1**. FDG-PET response evaluation, $SUV_{max}$ values and ΔTLG per patient.
**Supplementary table 2**. Response among patients with a CPS using a cutoff of 1, 5, and 10.
**Supplementary table 3**. Antibody panel used for imaging mass cytometry.
**Supplementary table 4**. Gene sets used in transcriptional analysis.

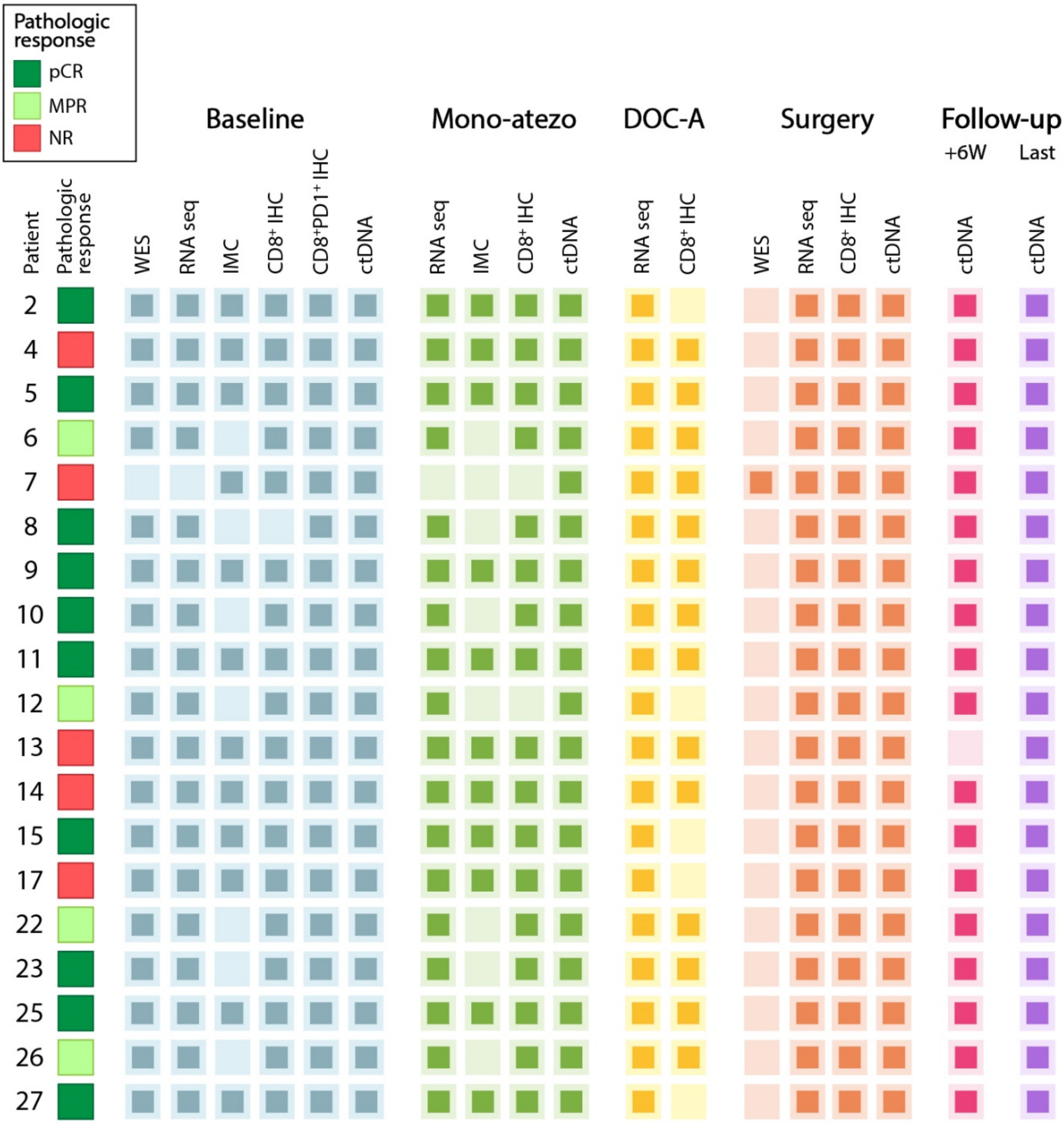


**Supplementary Figure 1 | Overview of samples used for translational analyses.** Dark squares indicate samples that were included in the analysis annotated per column. pCR: pathologic complete response; MPR: major pathologic response; NR: nonresponder; WES: whole exome sequencing; RNA seq: RNA sequencing; IMC: imaging mass cytometry; IHC: immunohistochemistry.

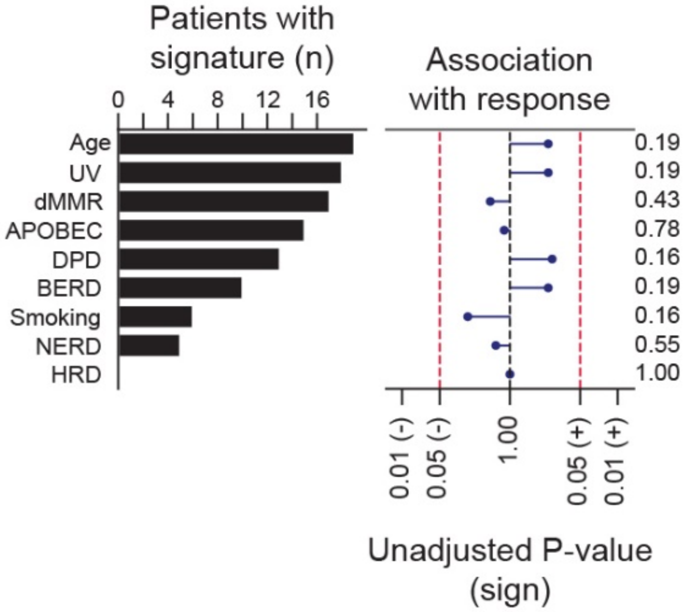


**Supplementary Figure 2 | Association of mutational signatures with response**
Barplot (left) of the number of patients (x-axis) with activity of specific mutational signatures (y-axis) and a lollipop plot (right) showing the signed P-value for association of mutational signature activity with response. Significance was tested using a two-sided Wilcoxon Signed-Rank sum test. X-axis on log10-scale.

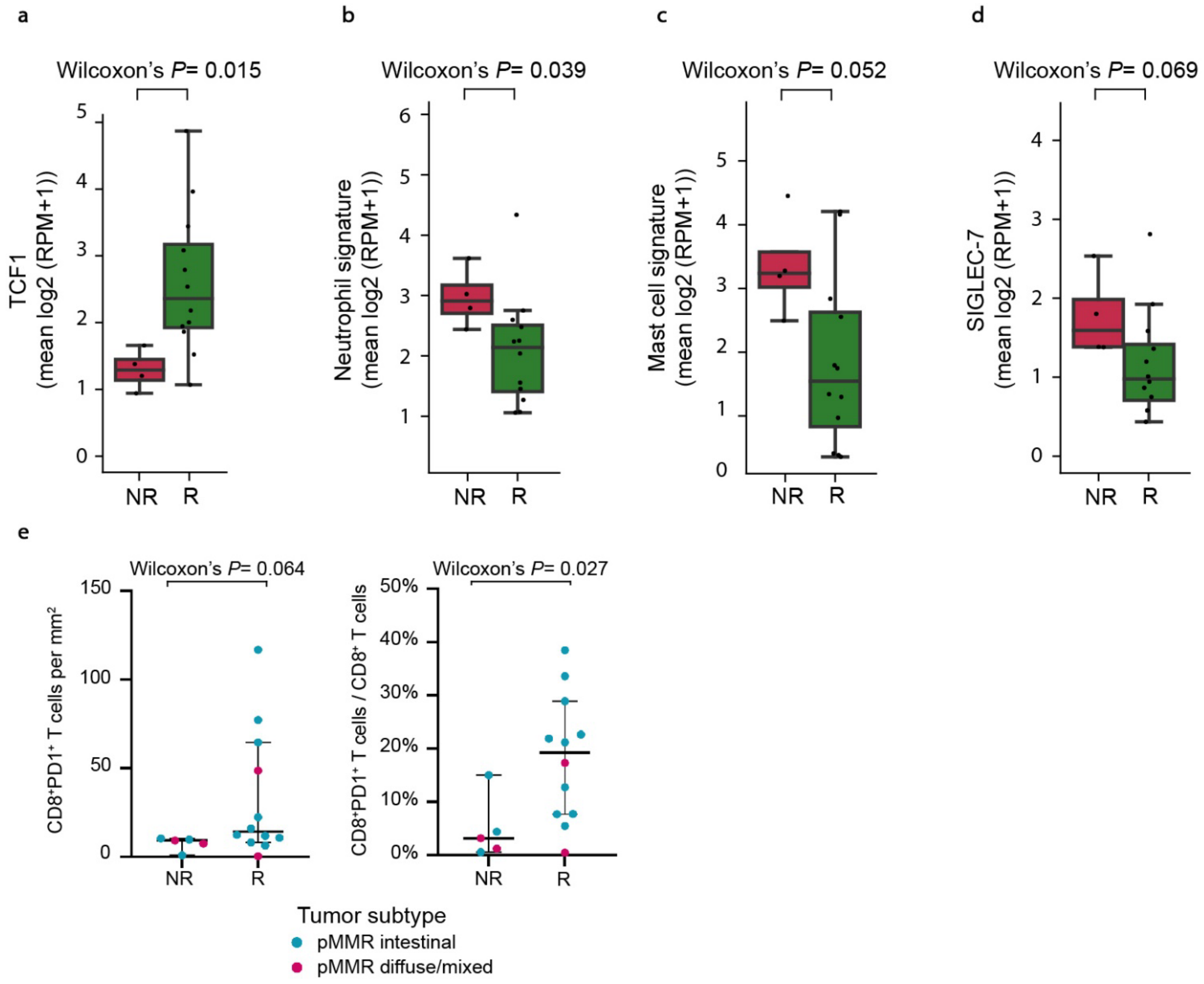


**Supplementary Figure 3 | Comparisons between pMMR responders and pMMR nonresponders of pretreatment *TCF1* and *SIGLEC-7* gene expression, neutrophil and mast cell signature plus immunohistochemistry for $CD8^+PD1^+$. a-d**, Pretreatment gene expression in nonresponders (NR, *n*=4) and responders (R, *n*=12) of **a**, *TCF1*. **b**, Neutrophil signature. **c**, Mast cell signature. **d**, *SIGLEC-7*. **a-d**, Boxplots represent the median, 25$^{th}$ and 75$^{th}$ percentiles; whiskers extend from the hinge to the largest value below 1.5 * IQR from the hinge. For comparison between NR and R, significance was tested using a two-sided Wilcoxon Rank-sum test. **e**, Pretreatment $CD8^+PD1^+$ T-cells using immunohistochemical double staining for CD8 and PD-1, shown per mm2 (left) and as percentage of all $CD8^+$ cells (right) in nonresponders (NR, *n*=5) versus responders (R, *n*=12). Dots represent individual patients and the colors represent the Lauren subtype (blue= intestinal type; purple= diffuse/mixed type). The horizontal line represents the median; whiskers show the 95% confidence interval. The difference between R and NR tested using a two-sided Wilcoxon Rank-sum test.

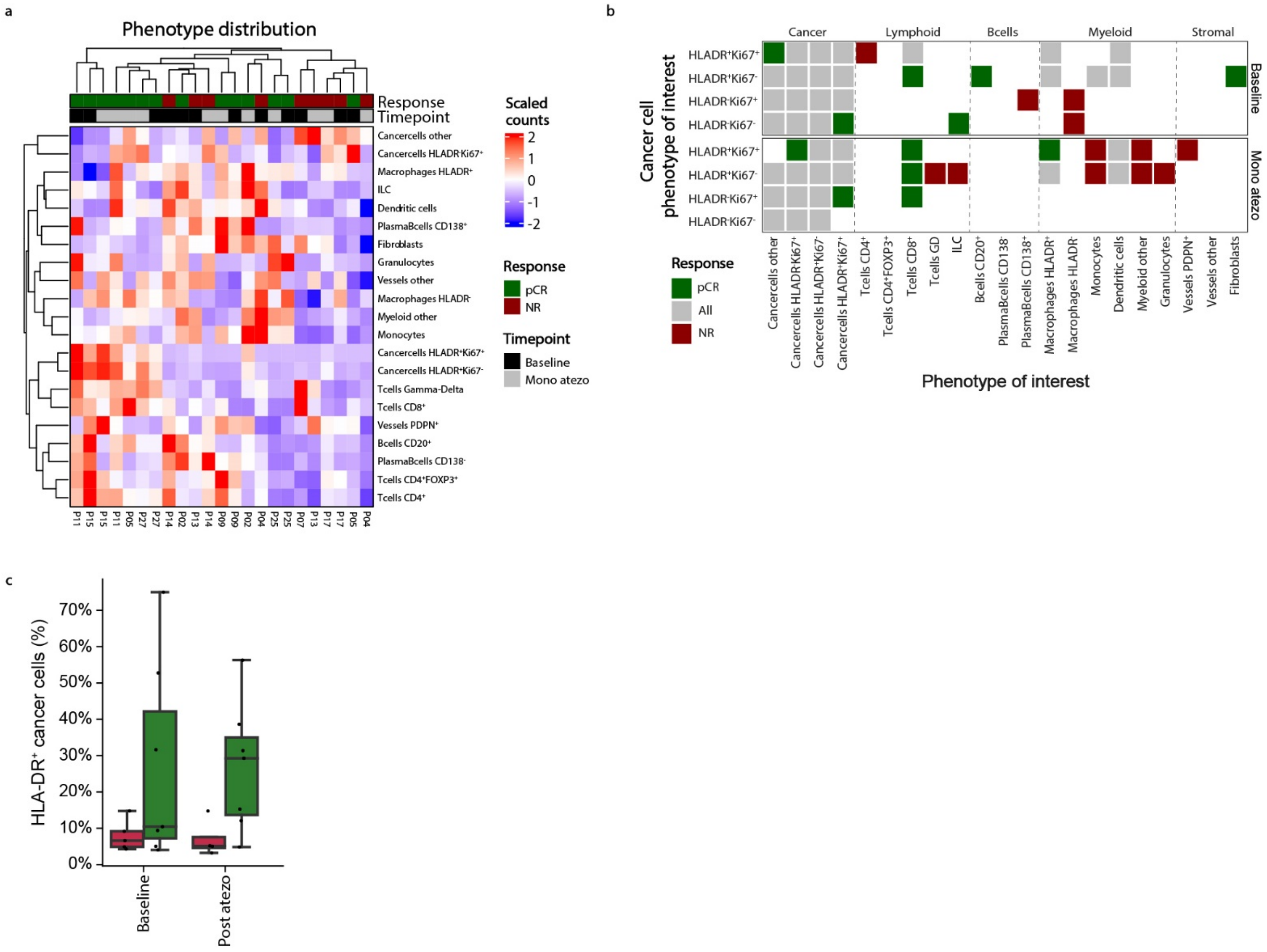


**Supplementary Figure 4 | Imaging mass cytometry of pMMR nonresponders and pMMR responders with a pCR. a**, Relative distribution of cellular phenotypes identified by IMC at baseline (pCR, *n*=7; NR, *n*=5) and after monotherapy atezolizumab (post atezo; pCR, *n*=7; NR, *n*=4). **b,** Spatial interactions between all populations types and cancer cell subsets. Interactions were considered specific if the colocalization of cell types was at least 2 z-scores above random cell distribution. Interactions specifically occurring in complete responders (pCR) are depicted in green, interactions specific for nonresponders (NR) in red and significant interactions that occurred in both groups are depicted in grey. **c,** Percentage of cancer cells expressing HLA-DR at baseline (pCR, green, *n*=7; NR, red, *n*=5) and after monotherapy atezolizumab (post atezo; pCR, green, *n*=7; NR, red, *n*=4). Boxplots represent the median, 25$^{th}$ and 75$^{th}$ percentiles; whiskers extend from the hinge to the largest value below 1.5 * IQR from the hinge. Dots represent individual patients.

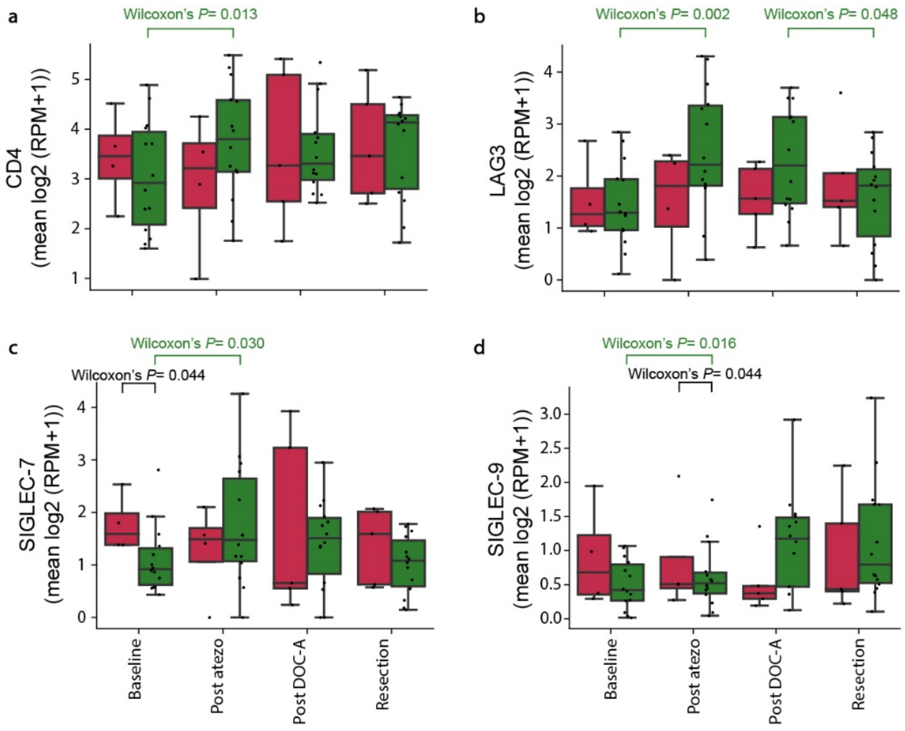


**Supplementary Figure 5 | Dynamics of CD4 T-cells, *LAG3*, *SIGLEC-7* and *SIGLEC-9* and comparison between responders and nonresponders.** Dynamics of gene expression of **a**, *CD4.* **b**, *LAG3*. **c**, *SIGLEC-7*. **d**, *SIGLEC-9*. **a-d**, Dynamics of gene expression in responders (R, green) and nonresponders (NR, red) at baseline (R, *n*=14; NR, *n*=4), post atezo (R, *n*=14; NR, *n*=4), post DOC-A (R, *n*=14; NR, *n*=5), and at resection (R, *n*=14; NR, *n*=5). Boxplots represent the median, 25$^{th}$ and 75$^{th}$ percentiles; the whiskers extend from the hinge to the largest value below 1.5 * IQR from the hinge. Differences between R and NR were tested using a two-sided Wilcoxon Rank-sum test. Differences between time points in R and NR separately were tested using a two-sided Wilcoxon Signed-Rank test. Significant p-values are depicted with accolades, colors indicate a significant increase/decrease in responders (green) or a significant difference between responders and nonresponders (black).

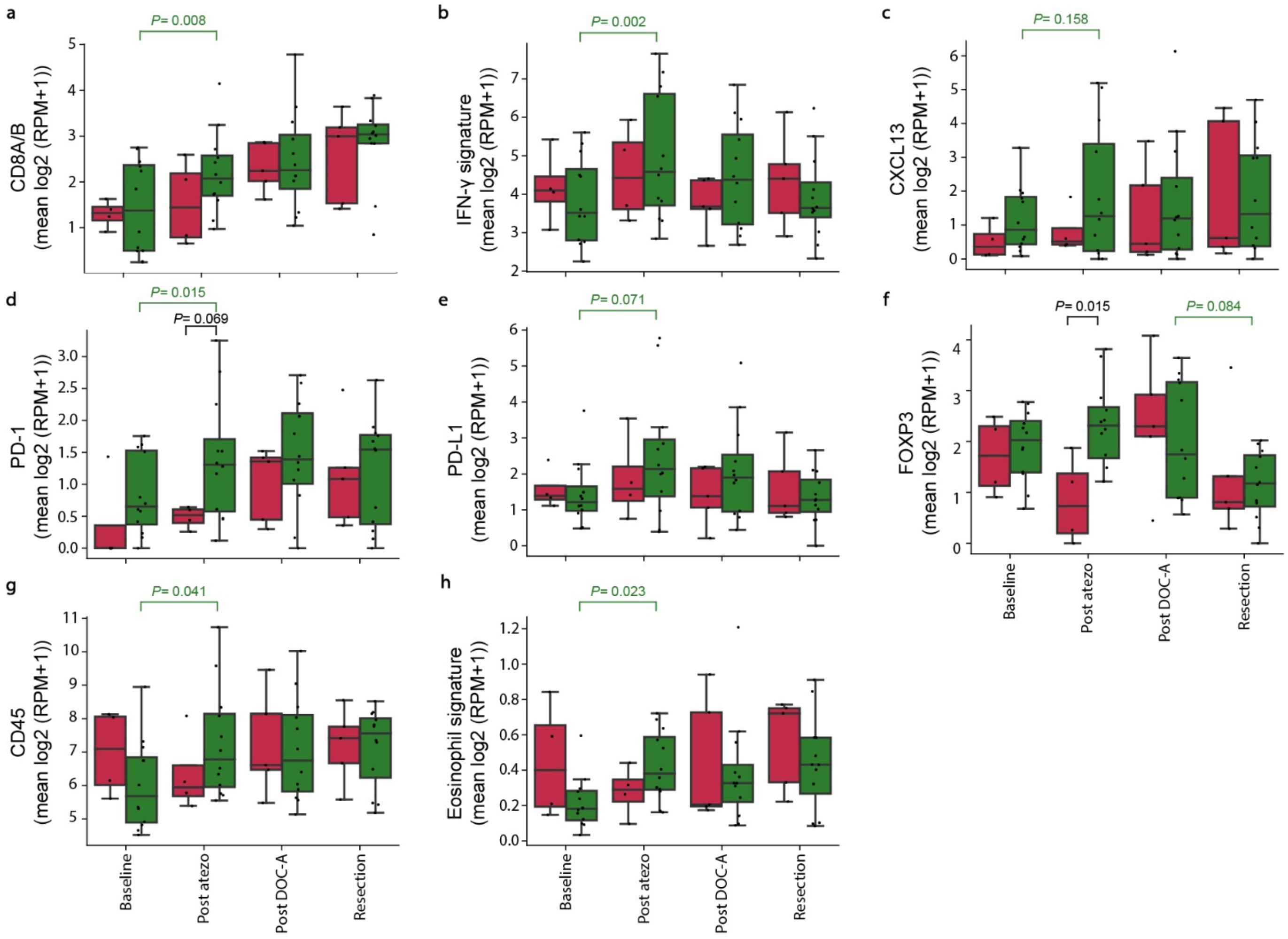


**Supplementary Figure 6 | Dynamics of immune-related gene expression in predicting response to ICB plus chemotherapy comparing pMMR responders and pMMR nonresponders. a-h**, Dynamics of gene expression of **a**, *CD8* (=*CD8A*+*CD8B*). **b**, IFN-γ signature. **c**, *CXCL13*. **d**, *PD-1*. **e**, *PD-L1*. **f**, *FOXP3*. **g**, *CD45*. **h**, Eosinophil signature. **a-h**, Dynamics of gene expression in pMMR responders (green) and pMMR nonresponders (red) at baseline (R, *n*=12; NR, *n*=4), after monotherapy atezolizumab (post atezo; R, *n*=12; NR, *n*=4), after DOC plus atezolizumab (post DOC-A; R, *n*=12; NR, *n*=5) and at resection (R, *n*=12; NR, *n*=5). Boxplots represent the median, 25th and 75th percentiles; whiskers extend from the hinge to the largest value below 1.5 * IQR from the hinge. Differences between R and NR were tested using a two-sided Wilcoxon Rank-sum test. Differences between time points in R and NR separately were tested using a two-sided Wilcoxon Signed-Rank test. Significant p-values are depicted with accolades, colors indicate a significant increase/decrease in responders (green) or a significant difference between responders and nonresponders (black).

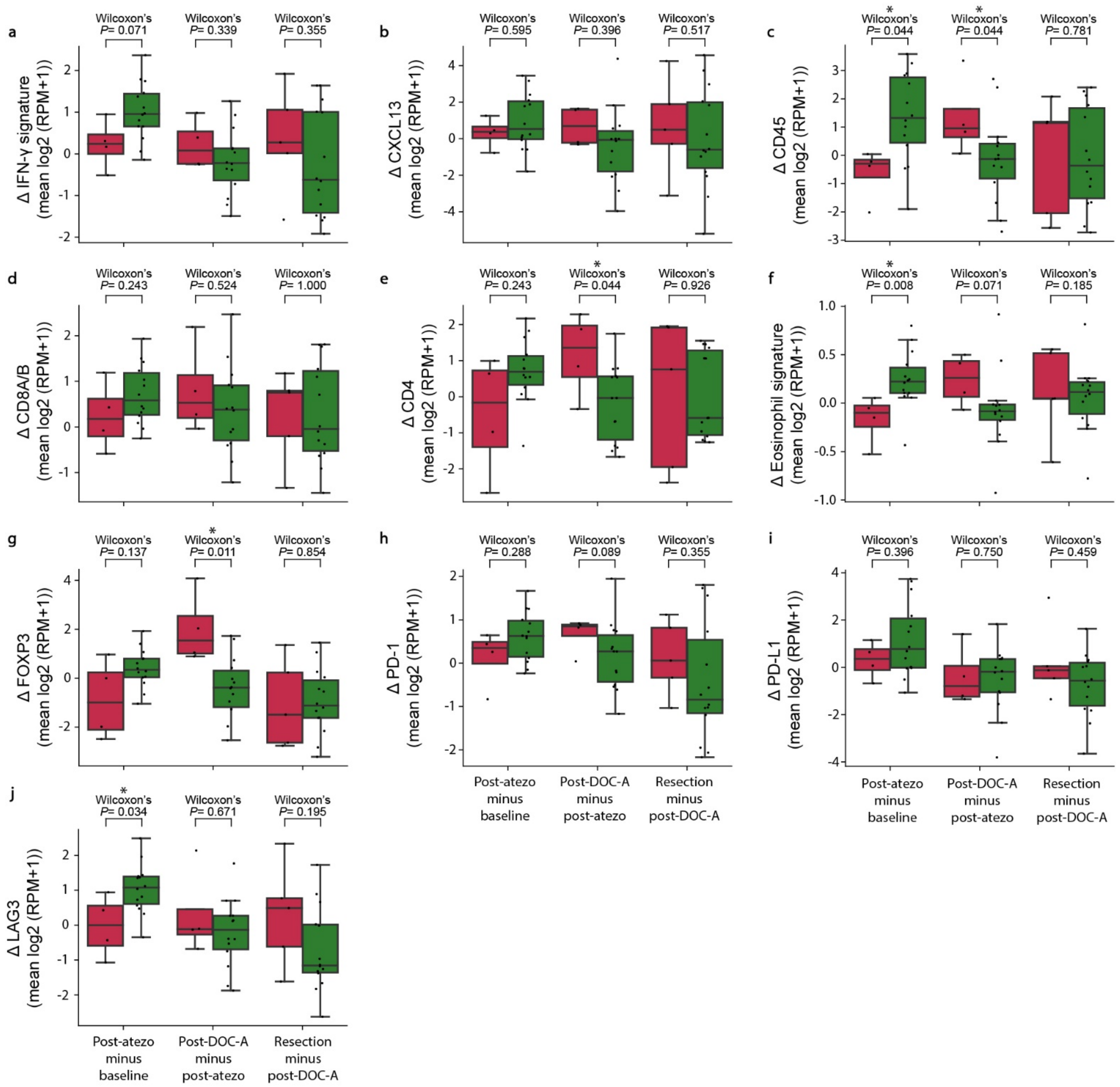


**Supplementary Figure 7 | Dynamics of immune-related gene expression presented as delta (Δ) values in responders and nonresponders. a**, Dynamics of gene expression or gene signature expression in responders (R, green) and nonresponders (NR, red) presented as delta (Δ) expression values of **a**, IFN-γ signature. **b**, *CXCL13*. **c**, *CD45*. **d**, *CD8* (= *CD8A/CD8B*) **e**, *CD4*. **f**, Eosinophil signature. **g**, *FOXP3*. **h**, *PD-1*. **i**, *PD-L1*. **j**, *LAG3*. **a-j**, Dynamics of gene expression represented as delta (Δ) expression values in responders (green) and nonresponders (red) at baseline, showing post atezo (R, *n*=14; NR, *n*=4) minus baseline (R, *n*=14; NR, *n*=4), post-DOC-A (R, *n*=14; NR, *n*=5) minus post atezo (R, *n*=14; NR, *n*=4), and resection (R, *n*=14; NR, *n*=5) minus post-DOC-A (R, *n*=14; NR, *n*=5). Boxplots represent the median, 25th and 75th percentiles; the whiskers extend from the hinge to the largest value below 1.5 * IQR from the hinge. The difference between R and NR was tested using a two-sided Wilcoxon Rank-sum test. All p-values are depicted with an accolade and significant value are identified with an asterisk.

**Supplementary table 1 | FDG-PET response evaluation, $SUV_{max}$ values and ΔTLG per patient.**

| **Patient** | **Response evaluation on FDG-PET** | **$SUV_{max}$ baseline** | **$SUV_{max}$ posttreatment** | ΔTLG |
|---|---|---|---|---|
| 1 | PR | 17.7 | 5.8 | -100% |
| 2 | CR | 9.0 | 3.4 | -100% |
| 4 | PR | 11.4 | 5.8 | -100% |
| 6 | Near-CR | 4.8 | 2.3 | -100% |
| 8 | Near-CR | 9.8 | 4.5 | -100% |
| 9 | PR | 20.8 | 6.5 | -100% |
| 10 | Near-CR | 6.9 | 4.7 | NE |
| 11 | Near-CR | 34.0 | 3.9 | -100% |
| 13 | SD | 4.7 | 5.7 | NE |
| 14 | PR | 10.9 | 4.5 | -100% |
| 15 | CR | 15.7 | 4.7 | -100% |
| 17 | Near-CR | 5.8 | 4.7 | NE |
| 22 | CR | 9.4 | 2.8 | -100% |
| 23 | Near-CR | 10.6 | 4.6 | -100% |
| 25 | CR | 5.9 | 2.5 | -100% |
| 26 | SD | 4.0 | 5.1 | NE |
| 27 | CR | 8.7 | 3.7 | -100% |

This table shows FDG-PET results per patient, including response evaluation, maximum standardized uptake value ($SUV_{max}$) at baseline and posttreatment as well as the difference in total lesion glycolysis (ΔTLG) between baseline and posttreatment FDG-PET scan.
PR: partial response; CR: complete response; NE: FDG-PET scan not evaluable for ΔTLG

**Supplementary table 2 | Response among patients with a CPS using a cutoff of 1, 5, and 10.**

| | **PD-L1 CPS <1 (*n* = 2)** | **PD-L1 CPS ≥1 (*n* = 18)** | ***P*-value *** |
|---|---|---|---|
| | | | 1.000 |
| **Responders** | 2 | 12 | |
| **Nonresponders** | 0 | 6 | |
| | **PD-L1 CPS <5 (*n* = 6)** | **PD-L1 CPS ≥5 (*n* = 14)** | ***P*-value *** |
| | | | 1.000 |
| **Responders** | 4 | 10 | |
| **Nonresponders** | 2 | 4 | |
| | **PD-L1 CPS <10 (*n* = 16)** | **PD-L1 CPS ≥10 (*n* = 4)** | ***P*-value *** |
| | | | 0.549 |
| **Responders** | 12 | 2 | |
| **Nonresponders** | 4 | 2 | |
| CPS = combined positive score; * P value was calculated using a two-sided Fisher's exact test. | | | |

**Supplementary table 3 | Antibody panel used for imaging mass cytometry.**

| Target | Clone | Lot | Company | Metal | Incubation time | Temp | Dilution |
|---|---|---|---|---|---|---|---|
| CD4 | EPR6855 | 1014578-6 | Abcam, Cambridge, United Kingdom | 145 Nd | Indirect ON | 4°C | 100 |
| TCRgd | H41 | D3021 | Santa Cruz biotechnology, Dallas, United states | 148 Nd | Indirect ON | 4°C | 25 |
| Anti-rabbit IgG | Polyclonal | GR3215731-15 | Abcam | 145 Nd | 1h | RT | 100 |
| Anti-mouse IgG | Polyclonal | GR3300461-1 | Abcam | 148 Nd | 1h | RT | 100 |
| CD8a | D8A8Y | 2 | Cell signaling technology, Danvers, United states | 146 Nd | 5h | RT | 50 |
| PD-1 | D4W2J | 1 | Cell signaling technology | 160 Gd | 5h | RT | 50 |
| ICOS | D1K2T(tm) | 4 | Cell signaling technology | 161 Dy | 5h | RT | 50 |
| CD204 | J5HTR3 | 2518439 | Thermo Fisher Scientific, Waltham, United States | 164 Dy | 5h | RT | 50 |
| CD103 | EPR4166(2) | GR3399209-2 | Abcam | 168 Er | 5h | RT | 50 |
| Tbet | 4B10 | B298378 | Thermo Fisher Scientific | 170 Er | 5h | RT | 50 |
| Caspase | D4V4B | 25 | Cell signaling technology | 172 Yb | 5h | RT | 50 |
| CD163 | D6U1J | 1 | Cell signaling technology | 173 Yb | 5h | RT | 50 |
| HLA-DR | TAL 1B5 | GR3424852-2 | Abcam | 141 Pr | 5h | RT | 100 |
| CD11b | D6X1N | 1 | Cell signaling technology | 144 Nd | 5h | RT | 100 |
| Granzyme B | D6E9W | 7 | Cell signaling technology | 150 Nd | 5h | RT | 100 |
| CD138 | 5A1E | 1 | Cell signaling technology | 155 Gd | 5h | RT | 100 |
| CD39 | EPR20627 | GR3274485-6 | Abcam | 157 Gd | 5h | RT | 100 |
| VISTA | D1L2G(TM) | 7 | Cell signaling technology | 158 Gd | 5h | RT | 100 |
| CD14 | D7A2T | 2 | Cell signaling technology | 163 Dy | 5h | RT | 100 |

| | | | | | | | |
|---|---|---|---|---|---|---|---|
| CD56 | E7X9M | 2 | Cell signaling technology | 167 Er | 5h | RT | 100 |
| CD7 | EPR4242 | GR3424737-2 | Abcam | 174 Yb | 5h | RT | 100 |
| CD11c | EP1347Y | GR3357092-17 | Abcam | 176 Yb | 5h | RT | 100 |
| CD45 | D9M8I | 12 | Cell signaling technology | 149 Sm | Overnight | 4°C | 50 |
| CD3 | EP449E | GR3418069-6 | Abcam | 153 Eu | Overnight | 4°C | 50 |
| PD-L1 | E1L3N(R) | 2 | Cell signaling technology | 156 Gd | Overnight | 4°C | 50 |
| FOXP3 | D608R | 2 | Cell signaling technology | 159 Tb | Overnight | 4°C | 50 |
| CD27 | EPR8569 | GR3446729-2 | Abcam | 175 Lu | Overnight | 4°C | 50 |
| Vimentin | D21H3 | 1 | Cell signaling technology | 194 Pt | Overnight | 4°C | 50 |
| Keratin | C11 and AE1/AE3 | 2 | Cell signaling technology | 198 Pt | Overnight | 4°C | 50 |
| TGFb | D10A8 | 157850 | Cell signaling technology | 89Y | Overnight | 4°C | 100 |
| CD20 | H1 | 1209781 | BD Biosciences, Franklin Lakes, United states | 142 Nd | Overnight | 4°C | 100 |
| CD68 | D4B9C | 2 | Cell signaling technology | 143 Nd | Overnight | 4°C | 100 |
| CD31 | 89C2 | 1 | Cell signaling technology | 147 Sm | Overnight | 4°C | 100 |
| CD57 | HNK-1 / Leu-7 | GR3373313 | Thermo Fisher Scientific, | 151 Eu | Overnight | 4°C | 100 |
| Ki-67 | 8D5 | 11 | Cell signaling technology | 152 Sm | Overnight | 4°C | 100 |
| IgG1 | D3W8G | 1 | Cell signaling technology | 154 Sm | Overnight | 4°C | 100 |
| IDO | D5J4E(TM) | 7 | Cell signaling technology | 162 Dy | Overnight | 4°C | 100 |
| CD45RO | UCHL1 | 1 | Cell signaling technology | 165 Ho | Overnight | 4°C | 100 |
| D2-40 | D2-40 | B316467 | Thermo Fisher Scientific, | 166 Er | Overnight | 4°C | 100 |
| CD38 | EPR4106 | GR3378690-1 | Abcam | 169 Tm | Overnight | 4°C | 100 |
| CD15 | MC480 | 5 | Abcam | 171 Yb | Overnight | 4°C | 100 |
| Bcatenin | D10A8 | 1 | Cell signaling technology | 196 Pt | Overnight | 4°C | 100 |
| Histone H3 | D1H2 | 1 | Cell signaling technology | 209 Bi | Overnight | 4°C | 50 |
| The location of each company its headquarter is provided in the first mention of the company in the table. Abbreviations: Temp: temperature; h: hour; RT: room temperature | | | | | | | |

**Supplementary table 4 | Gene sets used in transcriptional analysis.**

| Gene set | Number of genes | Gene symbols |
|---|---|---|
| B cells[81] | 9 | BLK, CD19, FAM30A, FCRL2, MS4A1, PNOC, SPIB, TCL1A, TNFRSF17 |
| CD8A/B[81] | 2 | CD8A, CD8B |
| Cytotoxic T-cells[82] | 6 | CTSW, GNLY, GZMA, GZMB, GZMH, PRF1 |
| Eosinophils[44] | 5 | SIGLEC8, RNASE2, RNASE3, IL5RA, CCR3 |
| Exhausted CD8 T-cells[81] | 4 | CD244, EOMES, LAG3, PTGER4 |
| IFN-γ signature (expanded)[36] | 18 | IDO1, CXCL10, HLA-DRA, STAT1, CD3D, CIITA, CD3E, CCL5, GZMK, CD2, CXCL13, IL2RG, NKG7, HLA-E, CXCR6, LAG3, TAGAP, GZMB |
| Macrophages[81] | 4 | CD163, CD68, CD84, MS4A4A |
| Mast cells[81] | 5 | CPA3, HDC, MS4A2, TPSAB1, TPSB2 |
| Neutrophils[81] | 7 | CEACAM3, CSF3R, FCAR, FCGR3B, FPR1, S100A12, SIGLEC5 |
| NK cells[82] | 2 | KLRF1, NCR1 |
| Regulatory T-cells[81] | 1 | FOXP3 |
| T-cells[81] | 6 | CD3D, CD3E, CD3G, CD6, SH2D1A, TRAT1 |
