# Supplementary material for: Neoadjuvant atezolizumab plus chemotherapy in gastric and gastroesophageal junction adenocarcinoma: the phase 2 PANDA trial
Source: Nat Med. 2024 Jan 8;30(2):519–30. doi: 10.1038/s41591-023-02758-x (PMC10878980; doi:10.1038/s41591-023-02758-x)
Supplement: Supplementary file 2 — Reporting Summary [file 41591_2023_2758_MOESM2_ESM.pdf]

nature portfolio

Corresponding author(s): M. Chalabi

Last updated by author(s): Dec 22, 2023

Reporting Summary

Nature Portfolio wishes to improve the reproducibility of the work that we publish. This form provides structure for consistency and transparency in reporting. For further information on Nature Portfolio policies, see our Editorial Policies and the Editorial Policy Checklist.

Statistics

For all statistical analyses, confirm that the following items are present in the figure legend, table legend, main text, or Methods section.

n/a	Confirmed	
☐	☒	The exact sample size (*n*) for each experimental group/condition, given as a discrete number and unit of measurement
☐	☒	A statement on whether measurements were taken from distinct samples or whether the same sample was measured repeatedly
☐	☒	The statistical test(s) used AND whether they are one- or two-sided<br>*Only common tests should be described solely by name; describe more complex techniques in the Methods section.*
☐	☒	A description of all covariates tested
☐	☒	A description of any assumptions or corrections, such as tests of normality and adjustment for multiple comparisons
☐	☒	A full description of the statistical parameters including central tendency (e.g. means) or other basic estimates (e.g. regression coefficient) AND variation (e.g. standard deviation) or associated estimates of uncertainty (e.g. confidence intervals)
☐	☒	For null hypothesis testing, the test statistic (e.g. *F*, *t*, *r*) with confidence intervals, effect sizes, degrees of freedom and *P* value noted<br>*Give P values as exact values whenever suitable.*
☒	☐	For Bayesian analysis, information on the choice of priors and Markov chain Monte Carlo settings
☒	☐	For hierarchical and complex designs, identification of the appropriate level for tests and full reporting of outcomes
☐	☒	Estimates of effect sizes (e.g. Cohen's *d*, Pearson's *r*), indicating how they were calculated

Our web collection on statistics for biologists contains articles on many of the points above.

Software and code

Policy information about availability of computer code

Data collection	TENALEA clinical trial data management system
Data analysis	R version 4.2.2 with the following packages: survival, survminer, coxphf, ggplot2 v3.3.6, mutSigExtractor 1.28 (https://github.com/UMCUGenetics/mutSigExtractor). R v4.2.0 with packages: ComplexHeatmap v2.14.0, ggplot v3.4.0.<br>Python 3 version 3.7.4 with Jupyter Notebook version 6.0.1, with the following packages: pandas 0.25.1; scipy 1.3.1; matplotlib 3.2.1; seaborn 0.9.0.<br>GraphPad Prism 9.0.2; HALOTM image analysis software 3.4.2986.185 (Indica Labs, Corrales, NM), QuPath 0.3.2; Qupath-extension-cellpose 0.5.1; Seqpurge 2019 09; bwa-mem 0.7.17; GATK 4.2; Mutect2 (tool in GATK); UmiAwareMarkDuplicatesWithMateCigar (tool in GATK); BaseRecalibrator (tool in GATK); dbSNP version 151 (tool in GATK); hisat2 2.1.0; Gensum (https://github.com/NKI-GCF/gensum).

For manuscripts utilizing custom algorithms or software that are central to the research but not yet described in published literature, software must be made available to editors and reviewers. We strongly encourage code deposition in a community repository (e.g. GitHub). See the Nature Portfolio guidelines for submitting code & software for further information.

Data

Policy information about availability of data

All manuscripts must include a data availability statement. This statement should provide the following information, where applicable:

- Accession codes, unique identifiers, or web links for publicly available datasets
- A description of any restrictions on data availability
- For clinical datasets or third party data, please ensure that the statement adheres to our policy

DNA and RNA sequencing data will be deposited into a repository on the European Genome archive (EGA) under EGAS00001007676, and these data will be made available for academic use upon reasonable request and within the confinements of the informed consent and in accordance with General Data Protection Regulation (GDPR).
The EGA accession number will be provided once this is available. The data are currently being uploaded.

Human research participants

Policy information about studies involving human research participants and Sex and Gender in Research.

Reporting on sex and gender	The study protocol did not include pre-specified sex- or gender-based analyses. Patients were included regardless of sex or gender.
Population characteristics	Eligible patients were at least 18 years with no maximum age (included patients were aged 46 to 76 years), were included regardless of sex and/or gender (included patients: 90% male, 10% female), and had previously untreated and primary resectable, histologically confirmed gastric or gastroesophageal junction adenocarcinoma without signs of distant metastases. All patients had an Eastern Cooperative Oncology Group performance status score of 0 or 1 and adequate hematologic and end-organ function. Key exclusion criteria were clinical symptoms or radiologic suspicion of perforation, active or history of autoimmune disease or immune deficiency, or history of malignancy within 3 years prior to screening (except malignancies with a negligible risk of metastasis or death).<br>The baseline characteristics of patients included in this trial are outlined in Table 1 of the manuscript.
Recruitment	Patients that presented with initial diagnosis of gastric/gastroesophagel junction cancer, either at our center or referred from other centers, and who were potentially eligible for this study were were informed of this and other studies for which they were eligible. patients who were deemed eligible were informed about the aims of the study, the possible adverse events, and the procedures and possible hazards to which he/she would be exposed. For patients with GEJ cancers for whom chemoradiotherapy was considered necessary to increase the chances of tumor-free resection margins, chemoradiotherapy was preferred after discussion in the multidisciplinary team (MDT).
Ethics oversight	This study was conducted in accordance with the International Conference on Harmonization (ICH) guidelines of Good Clinical Practice and the Declaration of Helsinki. The study protocol was approved by the Institutional Review Board of the NKI. All patients provided written informed consent before enrolment. There was no Data Safety Monitoring Board.

Note that full information on the approval of the study protocol must also be provided in the manuscript.

Field-specific reporting

Please select the one below that is the best fit for your research. If you are not sure, read the appropriate sections before making your selection.

☒ Life sciences ☐ Behavioural & social sciences ☐ Ecological, evolutionary & environmental sciences

For a reference copy of the document with all sections, see nature.com/documents/nr-reporting-summary-flat.pdf

Life sciences study design

All studies must disclose on these points even when the disclosure is negative.

Sample size	For this exploratory phase II study no formal sample size calculation was performed. The study aimed to recruit 20 patients to guarantee enough and timely accrual as well as evaluation of safety.
Data exclusions	Primary and secondary endpoints were analyzed in the per protocol population. All patients who did not receive at least one dose of study drugs (atezolizumab, docetaxel, oxaliplatin and capecitabine) were excluded from the per protocol population (n=1). For the translational analyses aiming to dissect the tumor microenvironment, patients who did not receive any of the intended treatment cycles of combination atezolizumab plus chemotherapy were excluded (n=1).
Replication	Replication is not applicable for clinical data. Translational experiments on human samples were not replicated due to limited material.
Randomization	Patients were not randomized, all patients received 1 cycle of atezolizumab (1200 mg) followed by 4 cycles of atezolizumab (1200 mg), capecitabine (850mg/m2), oxaliplatin (100 mg/m2) and docetaxel (50 mg/m2).

Blinding

No blinding was performed as all patients were allocated to receive 1 cycle atezolizumab followed by 4 cycles of atezolizumab, capecitabine, docetaxel, oxaliplatin and docetaxel.

Reporting for specific materials, systems and methods

We require information from authors about some types of materials, experimental systems and methods used in many studies. Here, indicate whether each material, system or method listed is relevant to your study. If you are not sure if a list item applies to your research, read the appropriate section before selecting a response.

Materials & experimental systems

n/a	Involved in the study
☐	☒ Antibodies
☒	☐ Eukaryotic cell lines
☒	☐ Palaeontology and archaeology
☒	☐ Animals and other organisms
☐	☒ Clinical data
☒	☐ Dual use research of concern

Methods

n/a	Involved in the study
☒	☐ ChIP-seq
☒	☐ Flow cytometry
☒	☐ MRI-based neuroimaging

Antibodies

Antibodies used

Immunohistochemistry (IHC):
• MLH1 Ready-to-use (=undiluted), M1, 6472966001, LotNo: g09887, Roche Diagnostics, Tucson, AZ, United States of America
• MSH2, Ready-to-Use (=undiluted), G219-1129, 5269270001, LotNo 2018: V0001229, LotNo 2019: V0001244, V0001272, V0001273, f06034, F1125, f17180. LotNo 2020: f15301, f19415, f29067. 2021 LotNo: g07854, g17138, g32360, h05779, h03163, h05779, h12642 Roche Diagnostics, Tucson, AZ, United States of America
• MSH6, 1/50 dilution, EP49, AC-0047, LotNo: 19060401, 20021305 , Abcam, Cambridge, United Kingdom
• Prior to August 2021: PMS2 Ready-to-use (=undiluted), clone EPR3947, 7604531 LotNo 2018: V0000986, V0001061, V0001217, V0001251, V0001198, V0001253, LotNo 2019: V0001253, F01588M, F060406M, F08243M, F09828M, F05846M, F07456M, LotNo 2020: F16520, F19021, F25056, G03551, G10164 2021: G21216, G10165, G05452, G32555, G33341, G19245,H000178, Roche Diagnostics, Tucson, AZ, United States of America
• Since August 2021: PMS2, 1/40 dilution, clone EP51, M3647, LotNo: 11286862, Agilent/DAKO, Santa Clara, CA, United States of America
• CD8 clone CD8/144B, 1/100 dilution, M710301-2, LotNo: 41311427, Agilent/DAKO, Santa Clara, CA, United States of America
• PD-1 clone CAL20, 1/250 dilution, ab237728, LotNo: GR3361014-6, AbCam, Cambridge, United Kingdom
• PD-L1 clone 22C3, 1/40 dilution, M365329-2, LotNo: 11295663, Agilent/DAKO, Santa Clara, CA, United States of America
Imaging mass cytometry (IMC):
• CD4 clone EPR6855, 1/100 dilution, metal: 145 Nd, LotNo: 1014578-6, Abcam, Cambridge, United Kingdom
• TCRgd clone H41, 1/25 dilution, metal: 148 Nd, LotNo: D3021, Santa Cruz biotechnology, Dallas, United states
• Anti-rabbit IgG, polyclonal, 1/100 dilution, metal: 145 Nd, LotNo: GR3215731-15, Abcam, Cambridge, United Kingdom
• Anti-mouse IgG, polyclonal, 1/100 dilution, metal: 148 Nd, LotNo: GR3300461-1, Abcam, Cambridge, United Kingdom
• CD8a clone D8A8Y, 1/50 dilution, metal: 146 Nd, LotNo: 2, Cell signaling technology, Danvers, United states
• PD-1 clone D4W2J, 1/50 dilution, metal: 160 Gd, LotNo: 1, Cell signaling technology, Danvers, United states
• ICOS clone D1K2T(tm), 1/50 dilution, metal: 161 Dy, LotNo: 4, Cell signaling technology, Danvers, United states
• CD204 clone J5HTR3, 1/50 dilution, metal: 164 Dy, LotNo: 2518439, Thermo Fisher Scientific, Waltham, United States
• CD103 clone EPR4166(2), 1/50 dilution, metal: 168 Er, LotNo: GR3399209-2, Abcam, Cambridge, United Kingdom
• Tbet clone 4B10, 1/50 dilution, metal: 170 Er, LotNo: B298378, Thermo Fisher Scientific, Waltham, United States
• Caspase clone D4V4B, 1/50 dilution, metal: 172 Yb, LotNo: 25, Cell signaling technology, Danvers, United states
• CD163 clone D6U1J, 1/50 dilution, metal: 173 Yb, LotNo: 1, Cell signaling technology, Danvers, United states
• HLA-DR clone TAL 1B5, 1/100 dilution, metal: 141 Pr, LotNo: GR3424852-2, Abcam, Cambridge, United Kingdom
• CD11b clone D6X1N, 1/100 dilution, metal: 144 Nd, LotNo: 1, Cell signaling technology, Danvers, United states
• Granzyme B clone D6E9W, 1/100 dilution, metal: 150 Nd, LotNo: 7, Cell signaling technology, Danvers, United states
• CD138 clone 5A1E, 1/100 dilution, metal: 155 Gd, LotNo: 1, Cell signaling technology, Danvers, United states
• CD39 clone EPR20627, 1/100 dilution, metal: 157 Gd, LotNo: GR3274485-6, Abcam, Cambridge, United Kingdom
• VISTA clone D1L2G(TM), 1/100 dilution, metal: 158 Gd, LotNo: 7, Cell signaling technology, Danvers, United states
• CD14 clone D7A2T, 1/100 dilution, metal: 163 Dy, LotNo: 2, Cell signaling technology, Danvers, United states
• CD56 clone E7X9M, 1/100 dilution, metal: 167 Er, LotNo: 2, Cell signaling technology, Danvers, United states
• CD7 clone EPR4242, 1/100 dilution, metal: 174 Yb, LotNo: GR3424737-2, Abcam, Cambridge, United Kingdom
• CD11c clone EP1347Y, 1/100 dilution, metal: 176 Yb, LotNo: GR3357092-17, Abcam, Cambridge, United Kingdom
• CD45 clone D9M8I, 1/50 dilution, metal: 149 Sm, LotNo: 12, Cell signaling technology, Danvers, United states
• CD3 clone EP449E, 1/50 dilution, metal: 153 Eu, LotNo: GR3418069-6, Abcam, Cambridge, United Kingdom
• PD-L1 clone E1L3N(R), 1/50 dilution, metal: 156 Gd, LotNo: 2, Cell signaling technology, Danvers, United states
• FOXP3 clone D608R, 1/50 dilution, metal: 159 Tb, LotNo: 2, Cell signaling technology, Danvers, United states
• CD27 clone EPR8569, 1/50 dilution, metal: 175 Lu, LotNo: GR3446729-2, Abcam, Cambridge, United Kingdom
• Vimentin clone D21H3, 1/50 dilution, metal: 194 Pt, LotNo: 1, Cell signaling technology, Danvers, United states
• Keratin clone C11 and AE1/AE3, 1/50 dilution, metal: 198 Pt, LotNo: 2, Cell signaling technology, Danvers, United states
• TGb clone D10A8, 1/100 dilution, metal: 89Y, LotNo: 157850, Cell signaling technology, Danvers, United states
• CD20 clone H1, 1/100 dilution, metal: 142 Nd, LotNo: 1209781, BD Biosciences, Franklin Lakes, United states
• CD68 clone D4B9C, 1/100 dilution, metal: 143 Nd, LotNo: 2, Cell signaling technology, Danvers, United states

	• CD31 clone 89C2, 1/100 dilution, metal: 147 Sm, LotNo: 1, Cell signaling technology, Danvers, United states<br>• CD57 clone HNK-1 / Leu-7, 1/100 dilution, metal: 151 Eu, LotNo: GR3373313, Thermo Fisher Scientific, Waltham, United States<br>• Ki-67 clone 8D5, 1/100 dilution, metal: 152 Sm, LotNo: 11, Cell signaling technology, Danvers, United states<br>• IgG1 clone D3W8G, 1/100 dilution, metal: 154 Sm, LotNo: 1, Cell signaling technology, Danvers, United states<br>• IDO clone D5J4E(TM) , 1/100 dilution, metal: 162 Dy, LotNo: 7, Cell signaling technology, Danvers, United states<br>• CD45RO clone UCHL1, 1/100 dilution, metal: 165 Ho, LotNo: 1, Cell signaling technology, Danvers, United states<br>• D2-40 clone D2-40, 1/100 dilution, metal: 166 Er, LotNo: B316467, Thermo Fisher Scientific, Waltham, United States<br>• CD38 clone EPR4106, 1/100 dilution, metal: 169 Tm, LotNo: GR3378690-1, Abcam, Cambridge, United Kingdom<br>• CD15 clone MC480, 1/100 dilution, metal: 171 Yb, LotNo: 5, Abcam, Cambridge, United Kingdom<br>• Bcatenin clone D10A8, 1/100 dilution, metal: 196 Pt, LotNo: 1, Cell signaling technology, Danvers, United states<br>• Histone H3 clone D1H2, 1/50 dilution, metal: 209 Bi, LotNo: 1, Cell signaling technology, Danvers, United states
Validation	Each IHC protocol has been developed and validated under standard operating procedures in a certified pathology lab (EN ISO15189, M258). Each new antibody lot is validated by testing multiple dilutions and evaluating them with the pathologist in a standardized method, using positive control tissues suitable for the antibody (images and protocol details available upon request). Antibodies were validated as described on the manufacturer's websites.<br><br>All IMC antibodies have been selected based on extensive validation for use in immunohistochemistry on FFPE tissue by the respective companies. All antibodies are tested in house on FFPE tonsil and colon tissue and staining patterns were compared to company datasheets and reported literature. After metal conjugation, the staining patterns of each antibody are once again validated by IHC and compared to staining prior to conjugation and reported literature. Furthermore, for each antibody, colocalization with expected other markers was confirmed by IMC.

Clinical data

Policy information about clinical studies

All manuscripts should comply with the ICMJE guidelines for publication of clinical research and a completed CONSORT checklist must be included with all submissions.

Clinical trial registration	ClinicalTrials.gov , NCT03448835
Study protocol	The study protocol will be made available upon request and will be uploaded together with initial submission of the manuscript.
Data collection	Patients were consulted in the outpatient clinic of the Netherlands Cancer Institute. Long-term follow-up was either performed at the outpatient clinic, by telephone or through telemedicine. Clinical data, including data regarding adverse events, were collected from time of signing informed consent until 100 days after the last administration of study drug. Outcome data and long-term survival data will be collected until five years after initial diagnosis. The first patient was enrolled on April 12th, 2018 and the last patient on May 14th, 2021. Data collection for the current analysis was performed on April 24th, 2023.
Outcomes	Primary endpoints were safety and feasibility of neoadjuvant capecitabine, oxaliplatin, docetaxel, and atezolizumab in gastric and gastroesophageal junction adenocarcinoma. Patients were closely monitored for adverse events (AEs) starting from signing informed consent until 100 days after last study medication, according to the Common Terminology Criteria for Adverse events (CTCAE) version 4.03. Safety was measured by (S)AEs and treatment-related complications leading to delays in systemic treatment and/or surgery past 9 weeks after start of the last treatment cycle. Additionally, the rate of anastomotic leakages after surgery, taking into account the type of surgical procedure, was closely monitored. The rate of anastomotic leakage was evaluated after the first 10 patients who underwent surgery; if <4 leakages had occured the study would continue to include 10 more patients. Feasibility was measured by adherence to the timelines of the study protocol.<br><br>Secondary and translational endpoints included disease-free survival, overall survival, radiologic tumor regression and efficacy, evaluated by histopathological response to treatment and associations between pathologic response and genomics, transcriptomics, and immunohistochemistry (IHC) and imaging mass cytometry (IMC) findings and circulating tumor DNA (ctDNA), including the TMB, gene expression signatures, and TCI.
